# Inferring Virus-Host relationship between HPV and its host *Homo sapiens* using protein interaction network

**DOI:** 10.1038/s41598-020-65837-w

**Published:** 2020-05-26

**Authors:** Qurat ul Ain Farooq, Zeeshan Shaukat, Tong Zhou, Sara Aiman, Weikang Gong, Chunhua Li

**Affiliations:** 1 College of Life Science and Bioengineering, Beijing University of Technology, Beijing, 100124 China; 2 Faculty of Information Technology, Beijing University of Technology, Beijing, 100124 China

**Keywords:** Computational biology and bioinformatics, Systems biology, Diseases, Pathogenesis

## Abstract

Human papilloma virus (HPV) is a serious threat to human life globally with over 100 genotypes including cancer causing high risk HPVs. Study on protein interaction maps of pathogens with their host is a recent trend in ‘omics’ era and has been practiced by researchers to find novel drug targets. In current study, we construct an integrated protein interaction map of HPV with its host human in Cytoscape and analyze it further by using various bioinformatics tools. We found out 2988 interactions between 12 HPV and 2061 human proteins among which we identified MYLK, CDK7, CDK1, CDK2, JAK1 and 6 other human proteins associated with multiple viral oncoproteins. The functional enrichment analysis of these top-notch key genes is performed using KEGG pathway and Gene Ontology analysis, which reveals that the gene set is enriched in cell cycle a crucial cellular process, and the second most important pathway in which the gene set is involved is viral carcinogenesis. Among the viral proteins, E7 has the highest number of associations in the network followed by E6, E2 and E5. We found out a group of genes which is not targeted by the existing drugs available for HPV infections. It can be concluded that the molecules found in this study could be potential targets and could be used by scientists in their drug design studies.

## Introduction

Human papilloma virus (HPV) is associated with approximately 5% of all human cancers affecting 0.6 million people worldwide with cervical, anal, oropharyngeal, penile and vulvovaginal cancers^1–3^. Among these cancers, cervical cancer ranks 4th in affecting women worldwide^4^ while in developing countries it ranks second^5^. According to World Health Organization (WHO) current factsheets, there are more than 100 genotypes of HPV, out of which 14 strains are high-risk. The most talked about high-risk HPV strains are HPV 6, 11, 16, 18, 31, 33, 35, 45, 52 and 58 with type 16 and 18 responsible for 70% of cervical cancer cases^6–8^. HPV is a serious threat to human life and it is causing 250,000 deaths annually, among which 85% of cases are occurring in low and middle-income countries^9^.

HPV is a small ~8 kb in size, non-enveloped circular dsDNA virus^5,10^. The HPV genome encodes 8 proteins among which 2 are structural viral capsid proteins (L1 and L2) while 6 are non-structural viral proteins (E1, E2, E4, E5, E6, E7)^10,11^. Besides these 8 proteins, there are a few other macromolecules found in literature which are actually the transcripts made by the fusion of two existing HPV proteins. E8∧E2, a transcript, is created by the fusion of E8 with carboxy terminal of E2^12^, and E1∧E4 is generated by the fusion of E1 to the Open Reading Frame (ORF) of E4^13^.

Protein interaction network provides a plethora of information when it comes to virus-host relationship because viruses entirely depend upon the host factors for their survival^14,15^. Viruses tend to regulate host biological processes by manipulating its cell proteome. Researchers have been using network biology for designing novel antiviral drug therapies^16^. We can dig deep down into the molecular mechanisms of the infections by analyzing the protein-protein interactions (PPIs) between the pathogen and its host. Researchers use various experimental and computational methods to identify PPIs and to study the ‘interactome’ of organisms.

A plethora of biological data has been generated because of the advent of “omics” data by high throughput technologies including genomics, proteomics and transcriptomics. In current postgenomic biomedical research, systems biology and network biology approaches are at the core and have been used by scientists to model disease networks for better understanding of the pathogen-host relationship and disease pathways^17–19^. By constructing pathogen-host interactome maps, we can find functionally important proteins and further study them by doing their functional enrichment analysis.

In current study, we integrate all the previous works on interactome of Human Papilloma virus to construct a unified and comprehensive protein interaction network of HPV with its host *Homo sapiens*. From small-scale experimental investigations to large-scale computational studies, we try to fetch every single interaction from the scientific literature. The study includes protein-protein interactions between HPV and Human, HPV and HPV identified by co-immunoprecipitation, mass spectrometry, Yeast 2 Hybrid, GST pull-down assays, tandem affinity purification, chromatin immunoprecipitation, indirect immunofluorescence assay and various other approaches. The data is also gathered from several well-established PPI databases including IMEx databases (DIP, HPIDB, BioGrid and IntAct) and databases which are not a part of IMEx consortium including VirHostNet and VirusMint. By constructing such a comprehensive interactome map of HPV with human, we will be able to study and understand the relationship between the pathogen with the host proteins and can find potential molecules involved in causing life-threatening infections and diseases. In a human body, every disease is triggered not just because of a single element but because of mutual interactions between various molecules.

The aim of this research is to find the molecules which are highly involved in provoking the HPV infections and to find out human proteins that are highly associated with viral proteins. We come up with certain host genes which are found to have distinct interactions with viral proteins and could be potential drug targets for scientists in their molecular docking studies.

## Results

### The HPV-Human protein interaction map and its statistical significance

**Figure 1:**
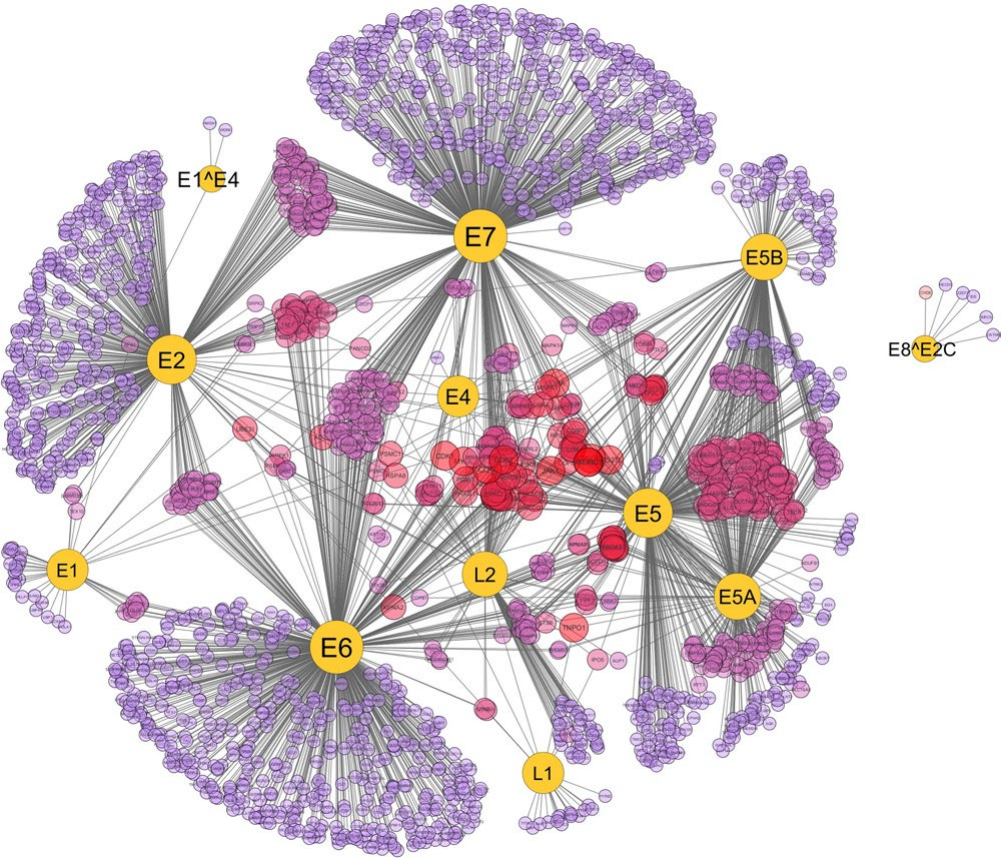
HPV-Human protein interaction network constructed in Cytoscape. The network comprises of 2073 nodes (proteins) with 2988 edges (interactions) between them. HPV proteins form highly connected hubs. The network is mapped according to node size. The lesser the number of interactions, the smaller the size of the node. HPV proteins E7 and E6 are of largest node size, indicating their substantial numbers of interactions. Viral proteins are represented in a uniform yellow color while human proteins in different colors. Host proteins with high numbers of interactions have larger node size (colored in bright red) compared to the proteins with lower numbers of associations (light purple).

**Figure 2:**
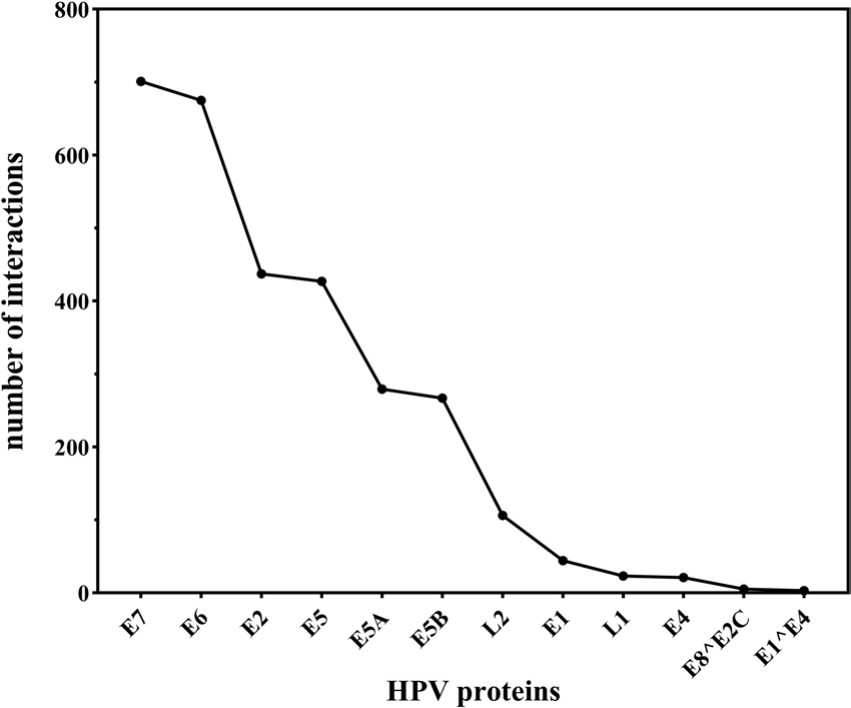
Numbers of interactions HPV proteins have in the overall network.

**Figure 3:**
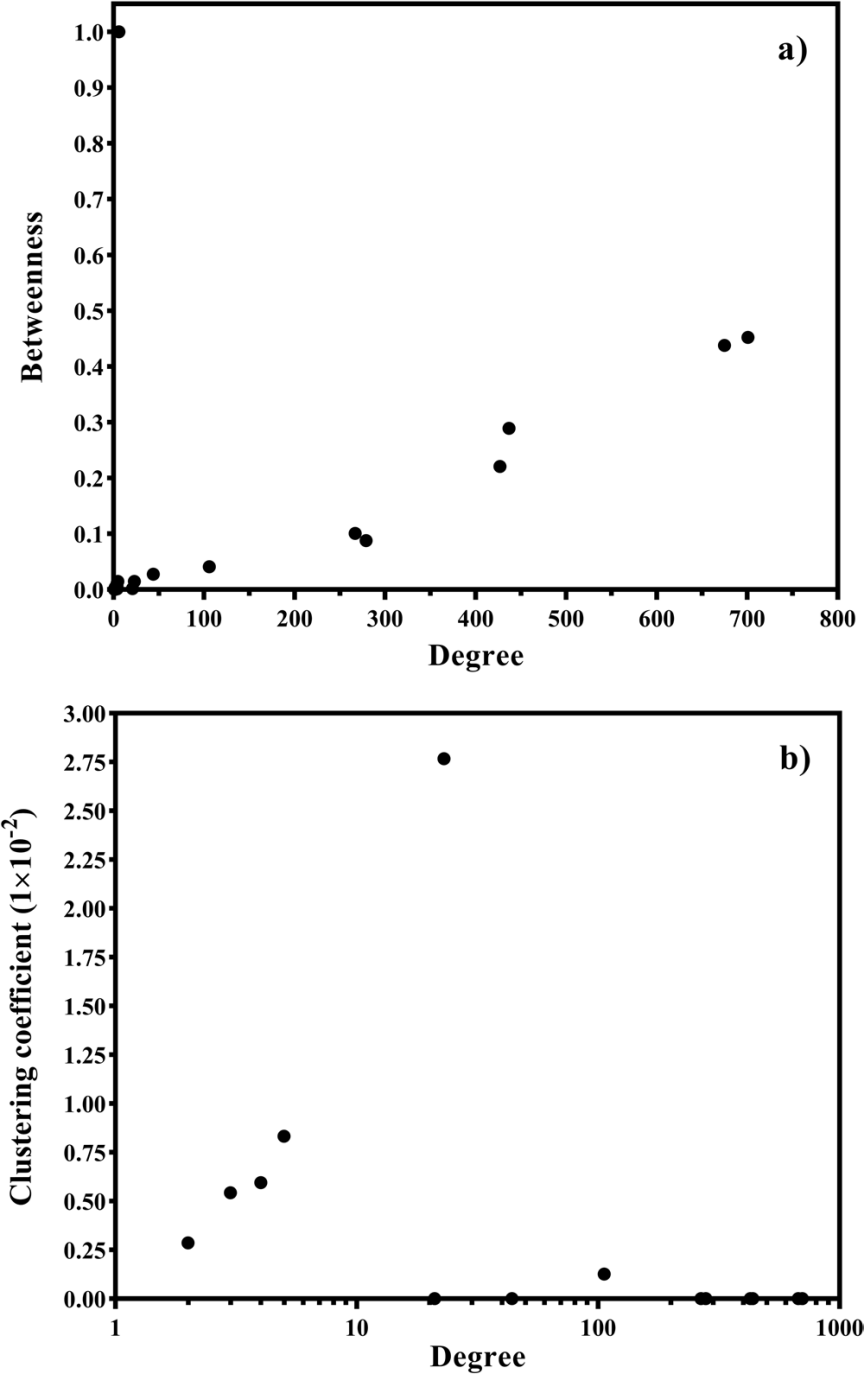
(**a**) Betweenness centrality and (**b**) clustering coefficient values of HPV proteins in the interaction network with the x-axis representing their degrees.

The integrated protein interaction network map of HPV with its host *Homo sapiens* constructed in Cytoscape is shown in Fig. 1. The network comprises of 2988 interactions between 12 HPV and 2061 human proteins (Supplementary table S1) in which highly associated proteins can be seen forming hubs in the overall network. The figure can be zoomed to see individual protein and its associations with other viral/host proteins in the network. It is noted that the interaction network of HPV with human is not much dense i.e. the average number of neighbors per node is 2.883 which means that the average number of interactions a node can have with the other nodes is not greater than 3, so the network density is less and it can be analyzed and explored easily. We can explore the network and dig deep down into the molecular associations of each protein in the network. The network was analyzed by using network analyzer tool of Cytoscape and we found out several important statistical features of the HPV-Human network including clustering coefficient, shortest path, average number of neighbors etc. Table 1 shows the important statistical measures of the network calculated by the network analyzer tool. The high scoring viral proteins are also mentioned in the table. CytoHubba is a built-in plugin of Cytoscape by which we can analyze hubs and even single nodes. By using this tool in our network, we found out that non-structural viral protein E7 is interacting with the most number of host proteins i.e. E7 has 701 associations in the overall network followed by non-structural proteins E6, E2 and E5 with a degree of 675, 437 and 427 respectively. Figure 2 represents a line graph showing the distribution of each HPV protein with their respective number of associations in the network. Figure 3 shows their betweenness centrality and average clustering coefficient graphs of the HPV-Human protein interaction network along with their average number of neighbors (degree). HPV is a virus and it is small in size, so the scatter plots are not very dense and is easily understandable.

**Table 1:** Statistics of the PPI network constructed between HPV-Human.

Attributes	Values
Num. of Nodes	2073
Num. of Edges	2988
Avg. Degree	2.883
High score interacting viral proteins	E7, E6, E2, E5
Clustering Coefficient	0.001
Network Density	0.001
Shortest Paths	4262192 (99%)

With its versatile and flexible layouts, Cytoscape helps user visualize and explore the macromolecular network in their best possible ways. Figure 1 also represents the analysis of the network and its visualization with respect to degree parameter. The node size is mapped according to its number of associations with other proteins in the network. The lesser the number of interactions, the smaller the size of the node while large size nodes represent proteins with high number of interactions. HPV oncoproteins E7 and E6 can be clearly seen having the largest node size, indicating their high number of associations with other proteins. Compared to viral proteins, the highest number of interactions a human protein has in the network is 5. MYLK, CDK7, CDK1, CDK2, JAK1, SQOR, RPS27L, MT-CO2, VKORC1, TNPO1 and COPA are the human proteins which are interacting with 5 viral proteins including oncoproteins E7 and E6 in the overall network. The enrichment analysis of these highly HPV-associated human genes was performed and is explained in the next section.

### Functional enrichment analysis

We performed pathway enrichment analysis of the top 25 host proteins scored and ranked by CytoHubba on the basis of Maximal Clique Centrality (MCC) which perceives on the concept that essential proteins tend to make clusters in a PPI network. Table 2 shows the pathway enrichment analysis results of the set of 25 human genes. The list of 10 enriched pathways is shown in the table together with their respective gene set size, p value and the value of corresponding False Discovery Rate (FDR). FDR^20^, also known as Benjamini-Hochberg (BH) method is a multiple-testing correction statistical method which uses the un-corrected p-value threshold and the number of tests to evaluate the fraction of falsely enriched pathways over the enriched pathways. The set of 25 genes ranked by CytoHubba on the basis of MCC was found to be enriched in vascular smooth muscle contraction, platelet activation, viral carcinogenesis, gap junction and several others mentioned in Table 2.

**Table 2:** Pathway enrichment analysis of the top 25 host genes ranked by CytoHubba on the basis of MCC.

Gene set	Description	Size	P value	FDR value
hsa04270	Vascular smooth muscle contraction	121	5.4 × 10^−6^	1.8 × 10^−3^
hsa04611	Platelet activation	123	1.4 × 10^−4^	2.3 × 10^−2^
hsa05203	Viral carcinogenesis	201	9.2 × 10^−4^	5.9 × 10^−2^
hsa04540	Gap junction	88	9.5 × 10^−4^	5.9 × 10^−2^
hsa04666	Fc gamma R-mediated phagocytosis	91	1.0 × 10^−3^	5.9 × 10^−2^
sa04658	Th1 and Th2 cell differentiation	92	1.1 × 10^−3^	5.9 × 10^−2^
hsa04914	Progesterone-mediated oocyte maturation	99	1.3 × 10^−3^	6.2 × 10^−2^
hsa04659	Th17 cell differentiation	107	1.7 × 10^−3^	6.8 × 10^−2^
hsa04110	Cell cycle	124	2.6 × 10^−3^	8.3 × 10^−2^
hsa04114	Oocyte meiosis	124	2.5 × 10^−3^	8.3 × 10^−2^

**Figure 4:**
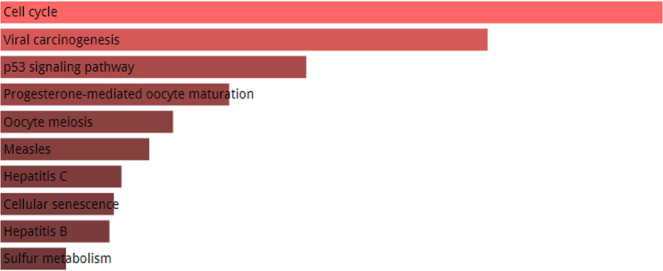
KEGG pathway analysis of top 11 highly HPV-associated human genes. The genes are a part of one of the chief cellular pathways i.e. cell cycle, viral carcinogenesis and p53 signalling pathway. The genes are also involved in several other pathways including progesterone-mediated oocyte maturation, oocyte meiosis, measles and HCV but the intensity of their involvement in these pathways is lesser. The length and color of the bar represent the intensity of the genes enriched in the pathway/process. The longer the bar and lighter the color, the more enriched the gene set in the specific pathway.

**Figure 5:**
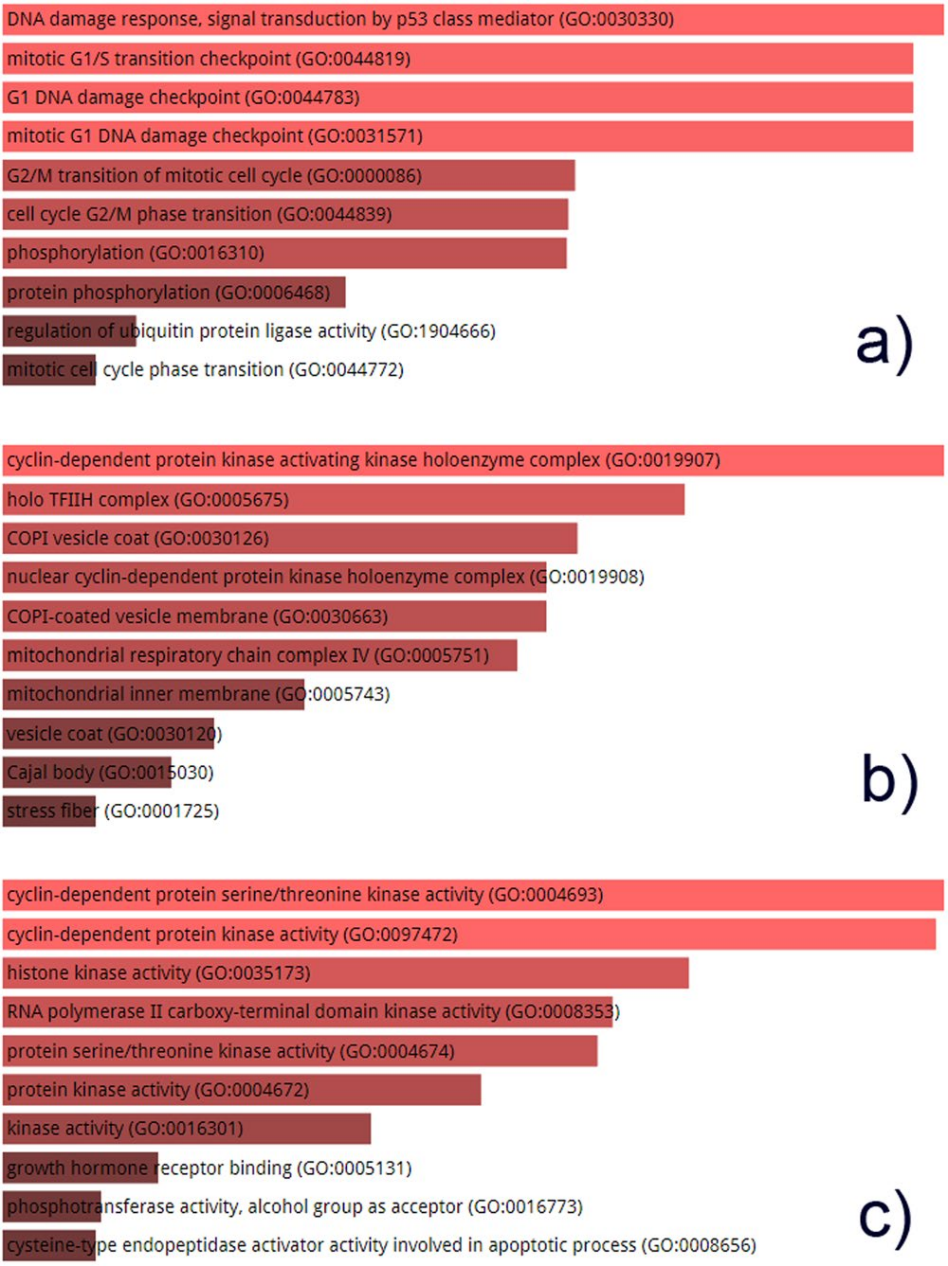
Gene Ontology of top 11 highly HPV-associated human genes. (**a**) The gene set is an important part of the DNA damage response which involves a cascade of processes activated by the p53 protein (GO:0030330), and also a part of cell cycle checkpoints including G1/S transition checkpoint (GO:0044819), G1 DNA damage checkpoint (GO:0044783), and a part of various other transition phases of cell cycle. (**b**) GO cellular component analysis reveals that the gene set is a major component of cyclin-dependent kinase activating kinase complexes responsible for cell cycle progression regulation (GO:0019907). The gene set is also a part of holo TFIIH complex (GO:0005675), a transcription factor essential for initiation of promoters. (**c**) GO molecular function analysis of the gene set shows that it is responsible for catalysis of a group of reactions and requires a CDK activity. The gene set is clearly involved in the regulation of cell cycle and the molecular functions they exhibit are cyclin-dependent protein serine/threonine kinase activity (GO:0004693), cyclin-dependent protein kinase activity (GO:0097472), histone kinase activity (GO:0035173) and several others as mentioned in the figure.

Additionally, we selected a group of 11 proteins with highest number of interactions in the HPV-Human protein interaction network and performed their pathway enrichment analysis and Gene Ontology. Among the group of proteins, each protein is interacting with 5 viral proteins in the network. KEGG knowledgebase lets us identify the pathways in which our input genes are over-represented and playing a pivotal role. KEGG pathway analysis of top 11 human proteins (Fig. 4) which have the highest number of interactions with HPV viral proteins reveals the fact that the gene set is actively involved in cell cycle which is one of the most important pathways for survival of an organism. The second most important pathway in which the gene set is actively playing a part is viral carcinogenesis, followed by p53 signaling pathway, progesterone-mediated oocyte maturation and so on. Gene Ontology enrichment analysis of a gene set reveals useful information regarding the biological processes the gene set is involved in, the molecular function of the genes and the cellular component of which the gene set is a part. Figure 5 shows GO biological process, cellular component and molecular function of the gene set having high connections with viral proteins.

We searched for the current drugs available to treat HPV infections on drug repurposing hub (https://clue.io/repurposing) and found out that the existing drugs Imiquimod, Podofilox and Trichloroacetic Acid target several proteins detected out in our study including TLR7, TUBA4A and TUBB respectively but there is no drug targeting the human proteins MYLK, CDK7, CDK1 and few more highly connected host proteins which are found to be having multiple associations with viral oncoproteins. We found out some drugs CaMKII-IN-1, BS 181 dihydrochloride, PF-573228, which will target the proteins MYLK, CDK7, and CDK1 respectively detected in our study but they are in their preclinical phases yet.

### Clustering analysis of the network

**Figure 6:**
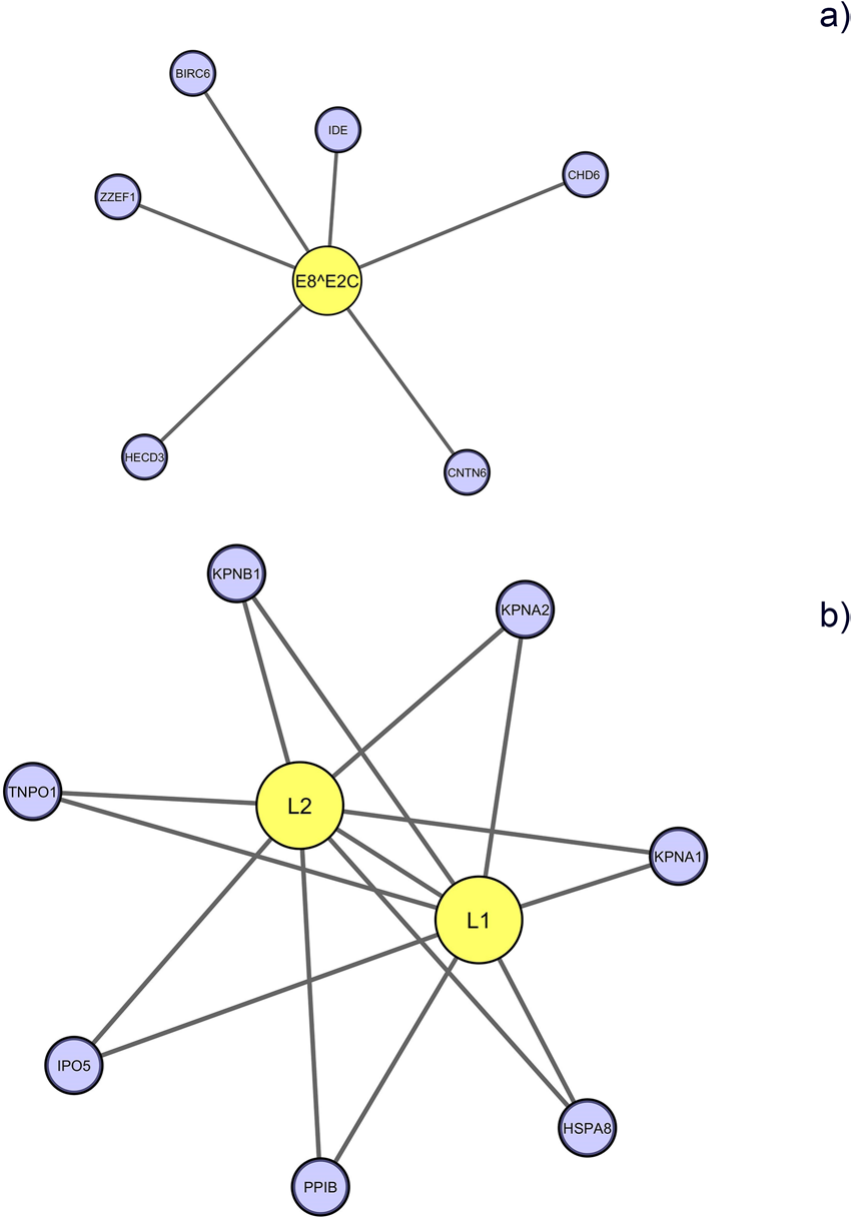
Clusters formed by CytoCluster using a hierarchical clustering algorithm. Viral proteins are represented as yellow nodes while human proteins are denoted in blue.

To find out protein complexes and functional modules in a protein-protein interaction network, we performed clustering analysis. Cytoscape has various clustering apps including CytoCluster^21^, ClusterMaker^22^, ClusterViz^23^, MCODE^24^ and ClusterOne^25^. All of these apps integrates different clustering algorithms based on different methods to analyze biological networks and identify crucial protein complexes. We used CytoCluster, which is a combination of six clustering algorithms with a mutual goal of identification of functional modules and protein complexes in the network. The clusters in the whole network were found to be making smaller sub-networks after clustering analysis performed by CytoCluster. One of the cluster (Fig. 6a) contains 7 nodes among which E8^E2C is a viral protein while ZZEF1, IDE, BIRC6, HECD3, CHD6, CNTN6 belong to human. Another cluster (Fig. 6b) consists of 9 nodes in which L1 and L2 are viral proteins while human proteins in the cluster are HSPA8, PPIB, KPNA2, TNPO1, KPNA1, KPNB1 and IPO5. The pathways in which these clusters are enriched are represented in Table S2.

## Discussion

The two most important pathways in which the set of human genes are involved are cell cycle and viral carcinogenesis which depicts the oncogenic nature of the Human papilloma virus proteins. The interactions of cell cycle-associated human genes with the oncoproteins of HPV give us an idea of how they might be involved in interfering the normal cell cycle and might alter the process which in turn can cause cancer. The third notable pathway in which the gene set is involved is p53 signaling pathway which is induced by many factors, some of which include activated oncogenes. Other disease pathways in which the gene set is found to be enriched include measles, hepatitis C and hepatitis B. The human genes which are highly associated with HPV and have maximum numbers of interactions with the viral proteins are: MYLK, CDK7, CDK1, CDK2, JAK1, SQOR, RPS27L, MT-CO2, VKORC1, TNPO1 and COPA. We integrated protein-protein interaction data from most pathogenic genotypes of HPV including HPV1, 3, 5, 6, 8, 9, 11, 16, 18, 31, 33 and HPV 39 from small-scale studies to large-scale extensive investigations carried out either experimentally or computationally.

Various researchers have been using network biology to study the disease and pathogen-host relationship on molecular level which ultimately helps in identifying key viral proteins and their human targets and helps scientists in further biological investigations. Kumar *et al*.^17^ studied the pathogenesis of *Leptospira interrogans* with Homo sapiens by analyzing their pathogen-host interactions after constructing a protein interaction network. Similarly, in our previous study, we constructed a comprehensive protein interaction map of HCV with its host human^26^ and then investigated it and found multiple potential drug targets. Besides infectious diseases, protein interaction networks have been used to inspect complex diseases including multiple sclerosis^27^, breast cancer metastasis^28^, Alzheimer’s disease^29^, HBV and hepatocellular carcinoma (HCC)^30^.

Previously, HPV-Human protein interaction network has been studied by other group of scientists too. Eckhardt *et al*.^31^ performed mass spectrometry analysis to find out interactions between HPV and human proteins. They identified 137 interactions between 9 viral proteins belonging to HPV genotype 31. On the other hand, our study presents an integrated network between multiple HPV genotypes and host proteins. We constructed a comprehensive protein interaction network and identified a total of 2988 interactions between 12 HPV and 2061 human proteins. We also incorporated the interactions identified by Eckhardt *et al*. in this study. Thus, our study presents a new approach of constructing a comprehensive protein interaction map of HPV with its host Homo sapiens by integrating all the previous small-scale and large-scale investigations performed on HPV PPI. We further have analyzed the PPI network by using various bioinformatics tools and have grouped the genes according to their most to least number of associations with human papilloma viral proteins and then performed KEGG pathway analysis to find out the biological pathways targeted by HPV. We also performed pathway enrichment of top 25 human genes ranked by CytoHubba on the basis of MCC to find out pathways they are enriched in along with their important statistical significance i.e. p-value and FDR. Additionally, we performed Gene Ontology analysis of the host genes which have highest number of associations with HPV proteins in the network to find out the biological processes targeted by HPV. We also found out that there are several human proteins which are currently not targeted by the available drugs available for HPV treatment. Some of the compounds targeting the high-connected proteins detected in this study are in their preclinical phases yet but there is no drug available currently to target COPA, TNPO1, RPS27L and SQOR, although they interact with multiple oncogenic HPV proteins.

## Conclusion

The infections caused by HPV are serious threats to human life. With emerging high throughput ‘omics’ technologies, we have huge amount of biological data which need to be investigated and analyzed by scientists from all over the world. Network biology is quite a new direction in the world of life sciences and it has been immensely used by researchers from past decade to unveil important information about pathogens, diseases and their impact on organisms. Our research concentrated on the protein-protein interactions between human papilloma virus and *Homo sapiens*. We constructed an integrated network of HPV with human and then analyzed it further using various techniques, which manifests potential target molecules. We are able to identity novel infection-related genes and further KEGG pathway and Gene Ontology enrichment analyses reveal the important biological pathways and processes targeted by the pathogenic infection at molecular level. In our research, we focus not only on protein-protein interactions from large database sources but also on individual interactions detected by scientists in their investigations. We are able to find out the involvement of target genes in several other disease pathways including measles, HCV and HBV. Though the intensity of their enrichment in these disease pathways is low but there is still a chance of knocking down these diseases by targeting these potential molecules.

## Methods

### Data collection

The first step of our study is to obtain a cutting edge picture of what is out there and how much has been done on protein interaction data and interactome of HPV with its host *Homo sapiens*. For this purpose, a PubMed advanced search is performed using multiple keywords. The search successfully yields 1222 results. The next step is to refine our search and to carefully analyze the studies specifically relating to protein-protein interaction (PPI) data of Human papilloma virus. Every study is carefully explored to get the maximum possible interactions between HPV and host proteins. From small-scale researches to extensive large-scale studies on protein-protein interactions involved in HPV and human, every possible interaction is gathered from the literature. Finally, 32 studies are chosen which contain exclusively protein-protein interaction data of HPV with its host and with its own viral proteins. The scientists used various potential protein-protein interaction detection methods and we gathered the information regarding every single interaction detected in their laboratories. Table 3 shows the list of selected studies performed on HPV-host protein-protein interactions (PINs). The list includes all the small-scale and large-scale investigations carried out either experimentally or computationally for detecting the PPIs.

**Table 3:** List of studies with accessible protein-protein interaction data of HPV-host.

No.	Paper	Num. of Interactions	Method of PPI detection	Ref
1	Gulati *et al*. (2019)	1	Mass spectrometry	^47^
2	Drews *et al*. (2019)	1	Co-transfection	^48^
3	Yang *et al*. (2019)	1906	HPIDB	^16^
4	Eckhardt *et al*. (2018)	137	Mass Spectrometry	^49^
5	DeSmet *et al*. (2018)	4	Co-immunoprecipitation, Mass Spectrometry	^50^
6	Sankovski *et al*. (2018)	1	Co-immunoprecipitation, Mass Spectrometry	^51^
7	Poirson *et al*. (2017)	47	Co-immunoprecipitation, GPCA	^52^
8	Spriggs *et al*. (2017)	3	Chromatin Immunoprecipitation (ChIP)	^53^
9	Dong *et al*. (2015)	877	IntAct, APID, VirHostNet	^32^
10	Tang *et al*. (2014)	1	Yeast 2 Hybrid, Co-immunoprecipitation	^54^
11	Jang *et al*. (2015)	253	Tandem affinity purification, Mass spectrometry	^55^
12	Kanginakudru *et al*. (2015)	1	Chromatin immunoprecipitation (ChIP)	^56^
13	Muller *et al*. (2012)	57	VirHostNet, VirusMint, PubMed	^57^
14	Woodham *et al*. (2012)	1	Co-immunoprecipitation, Electron Paramagnetic Resonance	^58^
15	Muller *et al*. (2012)	53	Yeast 2 Hybrid	^59^
16	Yaginuma *et al*. (2010)	1	Yeast 2 Hybrid, Co-immunoprecipitation	^60^
17	Xu *et al*. (2010)	1	Chromatin immunoprecipitation (ChIP)	^61^
18	Fertey *et al*. (2010)	1	Yeast 2 Hybrid, Co-immunoprecipitation	^62^
19	Côté-Martin *et al*. (2008)	1	Tandem affinity purification, Mass spectrometry	^63^
20	Wu *et al*. (2007)	2	GST pull down assay, Co-transfection	^64^
21	Zhang *et al*. (2005)	2	Immunoprecipitation, Transient transfection	^65^
22	Bernat *et al*. (2003)	1	Mammalian two-hybrid assay, Chloramphenicol acetyltransferase (CAT) reporter assay, GST pull-down assay and coimmunoprecipitation	^66^
23	Finnen *et al*. (2003)	1	Co-immunoprecipitation	^67^
24	Yang *et al*. (2003)	1	Immunoprecipitation,Indirect Immunofluorescence	^68^
25	Mantovani *et al*. (1999)	1	GST assay	^69^
26	Massimi *et al*. (1999)	1	Binding assays, Transient DNA replication assay, co-immunoprecipitation.	^70^
27	Thomas *et al*. (1999)	1	Co-transfection	^71^
28	Patel *et al*. (1999)	2	Co-immunoprecipitation, GST pull-down assay	^72^
29	Daniels *et al*. (1998)	2	Immunoprecipitation, Indirect immunofluorescence analysis	^73^
30	Swindle *et al*. (1998)	1	Co-immunoprecipitation	^74^
31	Jones et al. (1996)	1	Co-immunoprecipitation	^75^
32	Antinore et al. (1996)	4	Immunoprecipitation, Yeast two-hybrid system	^76^

The data gathered from these studies are merged and integrated, and after removing duplicates, 2988 unique interactions are found. Another challenge in this study is to bring all the data gathered from distinct studies into one common format, because for the network construction in Cytoscape, the data must be in one single format. Different scientists used different identifiers; some of them used protein/gene name while others used database identifiers e.g. UniProt, EMBL/GenBank or any other biological database identifier. For example, Dong *et al*.^32^ identified 877 protein-protein interactions between HPV and human proteins and he used UniProt identifiers in his large-scale study. To bring the data into a uniform format, an online UniProt ID Mapping tool^33^ is used to convert every single identifier into one common format which in our case is gene name.

### Construction and analysis of the virus-host PPI network in cytoscape

After having a uniform set of protein-protein interaction data, we use the latest version of Cytoscape (v3.7.1) to construct the integrated network between HPV and its host. Cytoscape^24^ is a most popular, user-friendly and freely available platform for the construction and exploration of biomolecular networks. Within the new version, it improves its performance in the context of versatility and interactivity for better analyses of the networks. Cytoscape has various tools and plugins by which the biomolecular networks can be analyzed and extensively explored on the basis of the attributes including degree distribution, betweenness centrality, clustering coefficient and so on. In current study, the network comprises of 2073 proteins (nodes) with 2988 Interactions (edges) between them. The number of human proteins in the network is 2061 while 12 proteins belong to HPV. The network is analyzed based on various topological measures including degree, diameter, betweenness centrality and clustering coefficient. Functionally important hubs are also explored which constitute the backbone of the HPV-Human protein interaction network.

- Degree of a node refers to the number of associations it has in the overall network, and it is one of the most basic properties of a network.
- Diameter of the network is the characteristic path length i.e. the shortest distance between two nodes in the network.
- Betweenness centrality is a notable parameter to identify essential and critical nodes in a network. It calculates the involvement of a node in the shortest paths in a network.
- Clustering coefficient determines the degree of the connectedness of the node’s neighbors.

### Network exploration and enrichment analysis

The next step in this study is to identify the pivotal human genes in the network and to perform their functional enrichment analysis. The set of genes which are interacting with multiple viral proteins should be playing a crucial part in the pathogenesis of the infections caused by HPV. Through functional enrichment analysis of the top-notch proteins, we can understand their molecular functions and can get to know the important biological processes they are involved in. Protein-protein interactions (PPI) are considered as unbiased data to explore the biological significance of gene sets, and have been used by many researchers for the investigation of gene sets^19,34,35^. Biological networks and pathways have been combined by various scientists in their analyses and they are proved to be successful in practice^36,37^. Enrichment analysis helps us to identify the pathways and biological processes in which a particular gene or gene set might be over-represented. The pathways are ordered on the basis of p value computed by a Fisher’s exact test ranging from 0 to 1 which accounts from highly significant to not significant, respectively. We first perform pathway enrichment analysis of top 25 host proteins selected on the basis of the scores computed by CytoHubba. CytoHubba^38^ is a novel Cytoscape app which ranks nodes in a network on the basis of network features. The score of every single node in the network is calculated by eleven network parameters which include degree, Maximum Neighborhood Component, Edge Percolated Component, Maximal Clique Centrality and so on. Through CytoHubba we can find the individual node scores including its degree, clustering coefficient, betweenness, and can make subgraphs of top-ranked nodes.

We also perform KEGG pathway enrichment analysis and Gene Ontology of the highly connected human proteins, filtered from the list of top 25 on the basis of their numbers of interactions with viral proteins in the pathogen-host protein interaction network. The online tool Enrichr^39^ (http://amp.pharm.mssm.edu/Enrichr), a comprehensive open-source web server, is used for gene set enrichment analysis. Enrichr is user-friendly and currently contains more than 100 gene set libraries with more than 180,000 gene sets. We can perform various analyses using this platform including KEGG (Kyoto Encyclopedia of Genes and Genomics), GO (Gene Ontology), Gene Expression Omnibus (GEO), Online Mandelian Inheritance In Man (OMIM) and several others. We will perform KEGG pathway analysis and GO analysis of the gene set which is associated with most number of viral proteins and has high interactions compared to the other host proteins from the network. The results obtained from Enrichr are based on the combined score of p-value and Z-score where p-value is computed using fisher exact test while z-score is computed as the deviation from the expected rank.

- KEGG^40,41^: KEGG, initially developed in 1995 is now an integrated knowledgebase for analyzing molecular datasets including genomics, metagenomics, proteomics, transcriptomics, metabolomics and other high-throughput biological data^42^. Through pathway enrichment analysis, we can identify the infectious pathways in which the gene set might be playing a crucial part.
- GO^43^: Gene Ontology is the most widely used and comprehensive knowledgebase containing over 7 million gene or gene product annotations of more than 3200 species. GO lets us identify the molecular function and subcellular localization of a gene product and also the biological process in which the gene product might be enriched or utilizing its molecular function in^44,45^.

Pathway enrichment analysis and Gene Ontology are at the core of bioinformatics for analyzing omics data generated by various genome-scale experiments, and with on-going research happening in this field, it is necessary to analyze macromolecular data with the same pace at which they are being produced by high-throughput methodologies. There are a lot of bioinformatics tools available to deal with the omics raw data and yield useful results.

To search for the current drugs available for the treatment of infections caused by HPV and to find out whether or not the existing drugs target the pivotal genes found out in current research, we use a next-generation drug library, the Drug Repurposing Hub^46^ which is a collection of more than 3000 clinical drugs targeting 2,247 proteins comprehensively annotated. It has comprehensive annotations of compounds and the information about clinical development status of drugs is available including the drugs that have been launched, or are in their pre-clinical stages or have been withdrawn from use along with the knowledge of their disease area.

## Supplementary information

Supplementary Information.
