# Supplementary material for: Inferring Virus-Host relationship between HPV and its host Homo sapiens using protein interaction network
Source: Sci Rep. 2020 May 26;10:8719. doi: 10.1038/s41598-020-65837-w (PMC7251128; doi:10.1038/s41598-020-65837-w)
Supplement: Supplementary file 1 — Supplementary Information. [file 41598_2020_65837_MOESM1_ESM.pdf]

Inferring Virus-Host relationship between HPV and its host *Homo sapiens* using protein interaction network

Qurat ul Ain Farooq[1], Zeeshan Shaukat[2], Tong Zhou[1], Sara Aiman[1], Weikang Gong[1], Chunhua Li[1]*

annie@emails.bjut.edu.cn, zee@emails.bjut.edu.cn, tongzhou8911@gmail.com, sara@emails.bjut.edu.cn, weikanggong@emails.bjut.edu.cn, chunhuali@bjut.edu.cn*

[1]College of Life Science and Bioengineering, Beijing University of Technology, Beijing 100124, China.

[2]Faculty of Information Technology, Beijing University of Technology, Beijing 100124, China.

*All correspondence should be addressed to Chunhua Li (E-mail: chunhuali@bjut.edu.cn)

Table S1: Protein-protein interactions between HPV and Human proteins integrated in this study

L1	DEFA5	E5A	DERL2	E6	USO1	E7	CHD4	E7	YJ005
E6	NHERF1	E5A	TECR	E6	PSMA5	E7	CRLF2	E7	DHSB
E1	DLG1	E5A	RAB32	E6	EFEMP2	E7	SMG7	L1	RL7L
E1	SUMO1	E5A	TMEM259	E6	LAMTOR3	E7	PUM1	E2	CTNB1
E1	POLA1	E5A	TMEM33	E6	SCNM1	E7	FAM3C	E2	APC
E1	PIAS1	E5A	SACM1L	E6	SRRM2	E7	NUMBL	E1	RFA1
E1	TRIP13	E5A	TMEM43	E6	IL18R1	E7	UBE2I	E7	YAP1
E1	SMARCB1	E5A	TMEM159	E6	VPS29	E7	RPL9	E7	AGAP1
E1	H1F0	E5A	RFTN1	E6	SMAD4	E7	ATP5B	E7	UBR2
E1	UBE2I	E5A	CALR	E6	TRAFD1	E7	PFKL	E1^E4	RFA1
E1	PIAS2	E5A	TMEM87B	E6	LAMTOR5	E7	IQGAP1	L2	HSP74
E1	IFIT1	E5A	SLC3A2	E6	TSEN34	E7	FXYD5	E2	PDIP2
E2	POLDIP2	E5A	BSG	E6	TCIRG1	E7	RPL3	E7	UGDH
E2	ZNF251	E5A	EMD	E6	IRF6	E7	DNAJB11	E6	PSB7
E2	TP53	E5A	TMX3	E6	FADD	E7	OGFOD1	E7	TFDP3
E2	TEFM	E5A	ATP2B4	E6	NDUFA2	E7	WDR5	E5	CD2A1
E2	RAB3IP	E5A	USMG5	E6	COPE	E7	HNRNPK	E5	CXA1
E2	MGA	E5A	RNFT1	E6	IRF3	E7	USP11	E5	RN126
E2	CHERP	E5A	TMEM120A	E6	NFX1	E7	CAST	E1	AN32E
E2	VPS39	E5A	AUP1	E6	CLIC1	E7	MBTPS1	E8^E2C	CNTN6
E2	TOB1	E5A	TAP2	E6	LRPPRC	E7	PCLO	E2	PCM1
E2	SRP68	E5A	RER1	E6	SYNGR2	E7	TRIM28	E5	VDAC3
E2	CEBPA	E5A	RAB18	E6	AGRN	E7	GAPDH	L1	KHDR1
E2	ZBTB38	E5A	RTN4	E6	CEP70	E7	RPL6	E2	FGFR1
E2	GPS2	E5A	S1PR3	E6	PUM2	E7	BIRC7	E2	FGFR2
E2	PLK2	E5A	SCFD1	E6	PI4KB	E7	CCNE1	E2	FGFR3
E2	BRD4	E5A	PIGT	E6	PTGES	E7	HIST1H2BK	E2	FGFR4
E2	EP300	E5A	RP2	E6	SNRPA	E7	SF3B1	E2	NM1
E2	CASP8	E5A	SLC25A12	E6	NCAPH	E7	TFDP1	E6	UBB
E2	TEX10	E5A	ABLIM3	E6	ERP44	E7	PTPN21	E6	LNX3
E2	BTBD2	E5A	SLC44A2	E6	CDC20	E7	PSMC2	E6	LNX4
E2	PMM2	E5A	TMED10	E6	PSMD14	E7	HNRNPAB	E6	MGRN1
E2	CDIPT	E5A	TRPA1	E6	BCAT2	E7	TUBA4A	E6	RNF25
E2	EZH2	E5A	ATRN	E6	TAX1BP3	E7	NOLC1	E6	RNF40
E2	NMI	E5A	ARL6IP5	E6	MGST3	E7	CCNA1	E6	TRAF5

E2	C1QBP	E5A	CD44	E6	TNPO2	E7	TUBA3C	E6	TRAF6
E2	NRBP1	E5A	TNPO1	E6	ELF1	E7	DTD1	E6	E6AP
E2	TIPIN	E5A	SLC35F5	E6	SPINT1	E7	NFIL3	E6	ITCH
E2	FZR1	E5A	NAT14	E6	TPP1	E7	TUFM	E6	AIMP2
E2	WWP2	E5A	SEC22B	E6	DNAJA1	E7	ZNF431	E6	TAX1BP1
E2	PPME1	E5A	IKBIP	E6	SFN	E7	SYPL1	E6	TRAF3IP2
E2	PSMA2	E5A	TM9SF3	E6	OTX1	E7	ISCU	E6	USP15
E2	UACA	E5A	IFITM3	E6	RBCK1	E7	MTA2	E7	UBB
E2	OAZ1	E5A	TMEM55A	E6	ICAM3	E7	HADH	E7	TRAF5
E2	BCL2L13	E5A	UBQLN1	E6	PFN2	E7	RBL2	E7	TRAF3IP2
E2	GGA1	E5A	TAP1	E6	SIPA1L1	E7	RBL1	E7	NEURL1
E2	RSF1	E5A	PSMC4	E6	LAMA5	E7	CCT4	E7	RNF135
E2	KRT81	E5A	AIFM1	E6	PPIA	E7	TFDP2	E7	SH3RF1
E2	SKP2	E5A	CAV1	E6	BCL2L13	E7	LAS1L	E7	TRAF2
E2	PTK2B	E5A	FADS2	E6	LAD1	E7	VIM	E7	TRAF3
E2	TAF6	E5A	MBOAT7	E6	YKT6	E7	CCT2	E7	TRAF4
E2	VPS52	E5A	ABHD12	E6	TBC1D7	E7	UBE2A	E7	TRIM9
E2	KAT7	E5A	RETREG3	E6	GIPC1	E7	PTRH2	E7	TRIM22
E2	ARFIP2	E5A	TRPV2	E6	GOPC	E7	TUBB2A	E7	TRIM32
E2	BCL2L1	E5A	UBAC2	E6	MCM5	E7	ZMYM3	E7	TRIM54
E2	POMP	E5A	SEC61A1	E6	CNIH4	E7	SQSTM1	E7	TRIM72
E2	TMF1	E5A	LPCAT3	E6	ZNF581	E7	KAT2B	E7	ZNF219
E2	KIF20A	E5A	CLPTM1	E6	PPL	E7	RARS2	E7	ZNF598
E2	LYST	E5A	SNX14	E6	CATSPER1	E7	EEF1A1P5	E7	BTBD15
E2	LCN2	E5A	RPN2	E6	TP53	E7	RPS27	E7	KCTD13
E2	SPOP	E5A	UBIAD1	E6	GPS2	E7	IRF1	E7	NACC1
E2	NR4A1	E5A	SLC4A2	E6	MED15	E7	SRP9	E7	SHKBP1
E2	BTBD1	E5A	GALNT5	E6	ERGIC3	E7	EIF4G1	E7	TNFAIP1
E2	TOX4	E5A	MT-ATP8	E6	HDGFL2	E7	BRCA1	E7	ZBTB9
E2	CDCP1	E5A	LPGAT1	E6	CTTNBP2NL	E7	MAP1S	E7	ZBTB20
E2	TOP1	E5A	SLC7A5	E6	MRPS16	E7	BIN3	E7	ZBTB32
E2	CEBPB	E5A	NDUFB1	E6	UBR5	E7	SPTBN1	E7	ZBTB42
E2	PRPF31	E5A	PLPP6	E6	CYLD	E7	RBBP7	E7	ZBTB43
E2	TAF1	E5A	SLC5A3	E6	WDR13	E7	PHC3	E7	ZBTB48
E2	AIDA	E5A	B4GAT1	E6	CARD9	E7	SNRPB2	E7	DCAF15
E2	SLC35B1	E5A	CTSB	E6	QARS	E7	RFC2	E7	MPND

E2	SF1	E5A	PON2	E6	PSMC1	E7	RPS25	E7	USP26
E2	PDIA3	E5A	TMCO3	E6	PSMD11	E7	ARID2	E7	USP29
E2	DDX56	E5A	SERINC1	E6	FAM216A	E7	SNAPC1	E7	USP33
E2	PCBP1	E5A	S100A13	E6	EMC9	E7	PDLIM7	E2	FANCD2
E2	TAX1BP1	E5A	ATP5J	E6	HDAC7	E7	SLC20A1	E7	FANCD2
E2	ENO1	E5A	RDH11	E6	SMAD3	E7	FAM208B	L2	FANCD2
E2	SCYL1	E5A	PLD3	E6	RCN2	E7	TSR2	L1	HSPA4L
E2	KRT6A	E5A	ATP5O	E6	STAG3L4	E7	CDK2	L1	HSPA1A
E2	DERL2	E5A	SAYSD1	E6	RNH1	E7	RIF1	L1	PPIB
E2	ZNF84	E5A	FITM2	E6	CCDC110	E7	TBP	L1	SDC2
E2	AREG	E5A	TMEM165	E6	FAM96B	E7	MOB4	L1	KPNA1
E2	ITGB4	E5A	PGRMC1	E6	PUM1	E7	LGALS14	L1	HSPH1
E2	NAP1L1	E5A	SLC27A4	E6	RPS27L	E7	MYC	L2	SGK1
E2	RACK1	E5A	RNF5	E6	PARVG	E7	AKAP8L	L2	KPNA3
E2	UBA1	E5A	SLC30A7	E6	SLC45A3	E7	P4HB	L2	CHEK1
E2	SRSF1	E5A	IMMT	E6	MAGI1	E7	TOB2	L2	MAP3K7
E2	HSPB1	E5A	ATP13A3	E6	ATP2A2	E7	TRIP13	L2	PAK3
E2	BAZ1A	E5A	HSD17B12	E6	NPM1	E7	AAK1	L2	TRIO
E2	SPTAN1	E5A	RETREG2	E6	GCN1	E7	RMDN3	L2	KAT7
E2	KAT2B	E5A	RMDN3	E6	PSMD1	E7	WDR77	L2	ABL1
E2	SMN1	E5A	OSTC	E6	DOCK7	E7	HM13	L2	PRKCG
E2	TBP	E5A	TM9SF2	E6	ORC4	E7	TAF15	L2	PRKCB
E2	EIF6	E5A	CSPG4	E6	CANT1	E7	CAD	L2	CDK1
E2	MSRB1	E5A	SQOR	E6	ANXA2	E7	DAZAP2	L2	CTSL
E2	GTF2B	E5A	ERLIN1	E6	YBX1	E7	LARP7	L2	CTSB
E2	CITED1	E5A	GALNT2	E6	SQOR	E7	TBL1XR1	L2	FURIN
E2	CCHCR1	E5A	NOTCH2	E6	RSRC2	E7	STK38	L2	ZBTB48
E2	CEP350	E5A	ERGIC1	E6	APMAP	E7	ZC2HC1C	L2	ARAF
E2	MAP1S	E5A	SRPRB	E6	MMP14	E7	ZER1	L2	HSPA8
E2	NFE2L2	E5A	SLC22A17	E6	SIRT6	E7	PTPN14	L2	CDK4
E2	TOPBP1	E5A	PDLIM7	E6	MMS19	E7	QKI	L2	ZNF250
E2	HOXC9	E5A	ITM2C	E6	EIF3L	E7	ELOB	L2	PRKACA
E2	RUNX2	E5A	RFT1	E6	PPP1R16B	E7	TTF1	L2	CDK11B
E2	SRSF7	E5A	APOB	E6	TRIM11	E7	TEAD4	L2	PRKACB

E2	AP3D1	E5A	HACD3	E6	ATP23	E7	NR1H4	L2	PPIB
E2	SPATS2	E5A	CDS2	E6	DNAJC25	E7	TAF6L	L2	JAK1
E2	CDC20	E5A	FAR1	E6	FN1	E7	E2F5	L2	CDK2
E2	HUWE1	E5A	WFS1	E6	VPS11	E7	C12orf10	L2	CTSS
E2	HSPA5	E5A	AFG3L2	E6	PSMC6	E7	FAM90A1	L2	MAPK3
E2	PDIA4	E5A	MAVS	E6	PSMD12	E7	SYT16	L2	MAPK1
E2	TRUB1	E5A	ERLIN2	E6	PSMC4	E7	ELOC	L2	AKT2
E4	HBA1;	E5A	MAIP1	E6	PSMC2	E7	NCOA1	L2	PRKCI
E4	SRPK1	E5A	GOLT1B	E6	CCT6A	E7	NUTM1	L2	ABL2
E4	KRT7	E5A	SLC35B2	E6	MTCL1	E7	NUMA1	L2	MAPK8
E5	TMEM161A	E5A	ZFPL1	E6	PSMC5	E7	TXNDC5	L2	MAPK9
E5	TECR	E5A	MPZL1	E6	TBP	E7	MATR3	L2	MAPKAPK2
E5	SLC7A5	E5A	MT-ATP6	E6	PSMD7	E7	PDIA4	L2	GSK3A
E5	SLC12A4	E5A	TMEM87A	E6	PSMD3	E7	ITPK1	L2	GSK3B
E5	TMED10	E5A	ACSL3	E6	RNPEP	E7	GATAD2B	L2	CDK7
E5	TMEM55A	E5A	TM9SF1	E6	PDLIM7	E7	HDAC7	L2	NEK2
E5	DDOST	E5A	YIPF4	E6	CCHCR1	E7	MBIP	L2	KPNA1
E5	YIPF3	E5A	TRPM4	E6	ABCE1	E7	CANT1	L2	LIMK1
E5	TAP2	E5A	GPAT4	E6	ZNF655	E7	UPP1	L2	DYNLT1
E5	RAB18	E5A	COPE	E6	RFC2	E7	EMC9	L2	CDK5
E5	SSR3	E5A	KDSR	E6	PSMA6	E7	E2F4	L2	CDK16
E5	TAP1	E5A	PBXIP1	E6	PSMD4	E7	ARHGAP35	L2	PRKCQ
E5	ICMT	E5A	ITGB1	E6	MCFD2	E7	SNW1	L2	PRKCD
E5	RHOT1	E5A	SSR3	E6	SHCBP1	E7	RB1	L2	MAPK7
E5	MTCH2	E5A	SCAMP3	E6	CCNY	E7	TRUB1	L2	PAK2
E5	TRPV2	E5A	CHP1	E6	EEF1A1P5	E7	CCT8	L2	STK3
E5	ABHD12	E5A	EI24	E6	STARD13	E7	SPCS2	L2	ROCK1
E5	IFITM3	E5A	ALG1	E6	INIP	E7	PCNA	L2	PRKG1
E5	PTTG1IP	E5A	COX20	E6	PTGES3	E7	POGZ	L2	IKBKE
E5	SACM1L	E5A	VKORC1L1	E6	NFU1	E7	BYSL	L2	MYLK

E5	ADCY6	E5A	MARCKS	E6	PSMB4	E7	GLYR1	L2	MAPK11
E5	TRAM1	E5A	PIGO	E6	EXOSC5	E7	UBR5	L2	CAMK4
E5	STOML2	E5A	SCD	E6	MAML1	E7	MRPS23	L2	MAPK6
E5	AMFR	E5A	UNC93B1	E6	PSMB1	E7	CDK1	L2	LRRK2
E5	TRPA1	E5A	FKBP8	E6	PSMB2	E7	CEP170	L2	OBSCN
E5	PCDH1	E5A	DIRC2	E6	ACTB	E7	CHD8	L2	CDC42BPA
E5	PREB	E5A	NDUFA4	E6	RAP2C	E7	CCT3	L2	MAST2
E5	CD47	E5A	ACSL4	E6	PSMB5	E7	RAN	L2	MARK2
E5	SLC16A3	E5A	FADS1	E6	PRTFDC1	E7	PSMC1	L2	TAOK1
E5	MPZL1	E5A	GPAA1	E6	ZNF417	E7	GTPBP10	L2	NEK9
E5	CDS2	E5A	ABCC4	E6	MORC4	E7	WDFY3	L2	KAT6B
E5	SEC11A	E5A	RPS27L	E6	UBE3A	E7	MFAP1	L2	SGK3
E5	SLC16A4	E5A	ENDOD1	E6	DUOX1	E7	RUVBL2	L2	AURKB
E5	TM9SF1	E5A	SLC16A3	E6	NOXA1	E7	COL27A1	L2	WNK4
E5	MICAL2	E5A	TMEM115	E6	PCBP1	E7	EXTL3	L2	TRIB3
E5	MGST1	E5A	ICMT	E6	SCO1	E7	CNP	L2	WNK3
E5	SLC35B2	E5A	CEPT1	E6	COX4I1	E7	TP73	L2	SLK
E5	ERLIN2	E5A	PIGU	E6	PLEKHA5	E7	IK	L2	TAOK3
E5	SLC2A1	E5A	CLCC1	E6	BCAS2	E7	ACTC1	L2	MKNK2
E5	SLC15A4	E5A	PRAF2	E6	CREBBP	E7	CCNA2	L2	NEK6
E5	MBOAT7	E5A	MTCH2	E6	MYC	E7	MGEA5	L2	PAK5
E5	GPAA1	E5A	ABCB7	E6	TNFRSF12A	E7	RACGAP1	L2	RPS6KB2
E5	ADGRA2	E5A	MRGPRF	E6	CKAP4	E7	SCD	L2	STK39
E5	SELENON	E5A	GGT7	E6	NME6	E7	PRPF31	L2	TBK1
E5	SRPRB	E5A	ATP9A	E6	VPS26A	E7	KBTBD4	L2	TNIK
E5	QPCTL	E5A	ASPH	E6	HADHA	E7	SCGB1A1	L2	MAP4K5
E5	ENDOD1	E5A	SLC38A10	E6	MYCBP2	E7	COPE	L2	CDC42BPB
E5	SLC35A2	E5A	FZD6	E6	COPS6	E7	HSPA5	E1	SLC9A3R1
E5	COPA	E5A	RAB34	E6	MYOZ1	E7	KLHL25	E1	MPDZ
E5	NDUFB1	E5A	STT3B	E6	ATP5L	E7	AKAP8	E1	PSMC1
E5	TNPO1	E5A	SCAMP4	E6	GADD45GIP1	E7	EEF1A1	E1	PSMC6
E5	S1PR3	E5A	CDIPT	E6	DMRT3	E7	PRMT5	E1	UBE2D2
E5	PLPP6	E5A	NCEH1	E6	PPP2R1A	E7	KCMF1	E1	UBE2L3

E5	SLC35E1	E5A	ARMCX3	E6	GNAI2	E7	COPA	E1	DLG4
E5	SEC22B	E5A	ATAD3A	E6	SHC1	E7	PHGDH	E1	TJP1
E5	DERL2	E5A	SPTLC2	E6	GABARAP	E7	PNPLA4	E1	PTPN13
E5	VKORC1	E5A	SNX19	E6	CD2BP2	E7	PRPH	E1	IL16
E5	IPO5	E5A	ATP2A2	E6	ZNF543	E7	PIN4	E1	SLC9A3R2
E5	ERLIN1	E5A	PLXNB2	E6	PAPLN	E7	SAP30BP	E1	MAGI2
E5	GALNT1	E5A	PHB2	E6	GLYCTK	E7	ZBTB25	E1	PPP1R9B
E5	PNPLA6	E5A	MYOF	E6	AES	E7	LMO4	E2	KPNA3
E5	PDGFRB	E5A	SPTLC1	E6	MPDU1	E7	RPS7	E2	APAF1
E5	AHNAK	E5A	TBL2	E6	SNX5	E7	RARA	E2	TRIM24
E5	ADGRG1	E5A	DNAJC13	E6	SLPI	E7	MEGF6	E2	TP73
E5	ATP5L	E5A	PGRMC2	E6	ANXA1	E7	NECAP2	E2	CFLAR
E5	ATP13A3	E5A	MT-CO2	E6	CDC42EP1	E7	UCK2	E2	PRPF4
E5	ABCC4	E5A	PIEZO1	E6	TK1	E7	ALPL	E2	STRN
E5	SVIP	E5A	SEC63	E6	MYCN	E7	ATP2C1	E2	TBL1X
E5	SYNGR1	E5A	GCN1	E6	MT1F	E7	FAM136A	E2	NR3C1
E5	PLIN3	E5A	COMT	E6	PPM1B	E7	MRPS12	E2	PGR
E5	PRAF2	E5A	CERS2	E6	SF3A3	E7	SPTLC1	E2	SP1
E5	SEC63	E5A	TMEM245	E6	TERT	E7	DNAJB1	E2	NR3C2
E5	TMEM126A	E5A	ABCD1	E6	TNPO1	E7	PDCD10	E2	HOXC4
E5	CPT1A	E5A	DERL1	E6	TFAP4	E7	POLR1C	E2	AR
E5	ITGB1	E5A	COPB1	E6	SRPRB	E7	XRCC4	E2	RARA
E5	STT3A	E5A	PTK7	E6	FTL	E7	SRP19	E2	THRA
E5	FAM8A1	E5A	SPNS1	E6	MT-ND1	E7	UNC119	E2	THRB
E5	CDIPT	E5B	TRAM1	E6	MT-ND2	E7	RNF31	E2	RXRA
E5	PLXNB2	E5B	STIM1	E6	MT-ND3	E7	PPIG	E2	PPARG
E5	YIPF4	E5B	TMEM259	E6	MT-ND4	E7	CD14	E2	PAFAH1B1
E5	KDSR	E5B	SRRM2	E6	EXOC8	E7	SIVA1	E2	NR2C2
E5	RABAC1	E5B	GLRB	E6	LGMN	E7	DAB2	E2	COPB1
E5	HSD17B2	E5B	RPL13A	E6	CEP57L1	E7	ZNF251	E2	COPA
E5	SCD	E5B	CHPF	E6	GALNT6	E7	EP300	E2	GNB1

E5	APOB	E5B	NIPSNAP1	E6	CTSD	E7	ERG	E2	GNB2
E5	ABCD1	E5B	RPS27L	E6	CCDC124	E7	PYGM	E2	UBE2I
E5	ATP2C1	E5B	RFTN1	E6	PHB2	E7	TAF4	E2	MDM2
E5	CLU	E5B	FKBP8	E6	NDUFS8	E7	VKORC1	E2	TLE1
E5	UNC93B1	E5B	STT3A	E6	NDUFB10	E7	IL18R1	E2	KHDRBS1
E5	SCAMP2	E5B	SLC30A7	E6	NDRG1	E7	NEFL	E2	PPARA
E5	ASPH	E5B	ECE1	E6	PSMA4	E7	TFAP2A	E2	BPTF
E5	TMCO1	E5B	SLC1A5	E6	PSMF1	E7	FCN1	E2	SRSF5
E5	SEC61B	E5B	PLD3	E6	PSMA1	E7	E2F3	E2	NR5A1
E5	MT-CO2	E5B	CNTNAP1	E6	IWS1	E7	CENPB	E2	CREBBP
E5	BDKRB2	E5B	LRRC8A	E6	PPP2R2A	E7	FOXK1	E2	KAT2A
E5	STOM	E5B	PTK7	E6	PLOD2	E7	MRPL36	E2	FBXW7
E5	ALG1	E5B	SGCB	E6	ANO8	E7	SLC1A3	E2	DDX11
E5	DAD1	E5B	P4HB	E6	PPP1R18	E7	UBR4	E2	EP400
E5	OXTR	E5B	RPL21	E6	ZHX2	E7	IKZF3	E2	PITX2
E5	ATP5C1	E5B	ADAM9	E6	SEC61A1	E7	MAP4	E2	TBL1XR1
E5	QSOX1	E5B	RPL3	E6	S100A14	E7	PARD6B	E2	BAZ2B
E5	NDUFA4	E5B	CDKAL1	E6	WRNIP1	E7	LOX	E2	FBXW11
E5	SCAMP3	E5B	FNDC3B	E6	NINJ1	E7	CHRNA5	E2	BTRC
E5	FAS	E5B	TMEM55A	E6	CCDC114	E7	MEOX2	E6	SGK1
E5	NDUFS1	E5B	DNAJA3	E6	TMEM87B	E7	SRI	E6	WWP2
E5	SFXN3	E5B	ABCD1	E6	VPS35	E7	PDLIM4	E6	ABLIM1
E5	HMOX2	E5B	ATP2A2	E6	P2RX5	E7	C1orf43	E6	DVL1
E5	TMEM120A	E5B	CPT1A	E6	GFPT1	E7	DNAH2	E6	MAP2K7
E5	ADAM9	E5B	MMP14	E6	SLC25A3	E7	MAGED1	E6	CHEK1
E5	TMEM33	E5B	EMD	E6	MCM3	E7	ATP5I	E6	TNFRSF10B
E5	PGRMC2	E5B	BSCL2	E6	CTSB	E7	SRRM2	E6	IKBKB
E5	MYOF	E5B	ATP2B1	E6	SOGA1	E7	SERPINF1	E6	AURKA
E5	FAF2	E5B	SLC27A3	E6	RUVBL2	E7	GABRA2	E6	CHUK
E5	ATP13A1	E5B	PNPLA6	E6	UBE2W	E7	CEP126	E6	TRIM24
E5	RAB34	E5B	PDIA3	E6	XRCC1	E7	SHC1	E6	TGFB1I1

E5	TMEM109	E5B	COPA	E6	MCM7	E7	PRDX6	E6	MAP3K7
E5	ATP9A	E5B	GPX8	E6	TSC22D3	E7	CCND3	E6	JAK2
E5	TOR4A	E5B	ERLEC1	E6	APH1A	E7	SUZ12	E6	LPXN
E5	FZD7	E5B	RHOT1	E6	UBN1	E7	ACOT9	E6	ROCK2
E5	FKBP8	E5B	COPG1	E6	PDIA3	E7	RBM4	E6	TADA3
E5	GGT7	E5B	PLPP6	E6	POLE3	E7	CCDC94	E6	MPDZ
E5	PTDSS2	E5B	FAF2	E6	HMGB1	E7	HSP90AA1	E6	ABL1
E5	ABHD16A	E5B	ABHD12	E6	DMD	E7	TAF9	E6	PRKCG
E5	CAV1	E5B	PKD2	E6	ST6GALNAC6	E7	RCOR1	E6	PRKCB
E5	GLIPR1	E5B	CANX	E6	HMGA1	E7	LMO2	E6	LYN
E5	CLPTM1	E5B	WLS	E6	LRRC59	E7	POLR2E	E6	NGFR
E5	ATP1A1	E5B	PTTG1IP	E6	METTL17	E7	GDI2	E6	IRF1
E5	HLA-B	E5B	MAP7D1	E6	PFKP	E7	GMCL1	E6	IL1R1
E5	STX4	E5B	ACSL3	E6	ITPKC	E7	AKAP10	E6	FER
E5	RPN2	E5B	VAPA	E6	NATD1	E7	PSTPIP1	E6	PRKACA
E5	ANO10	E5B	GAS6	E6	PTPN6	E7	MLST8	E6	EIF2AK2
E5	RPL13A	E5B	SCFD1	E6	RUNX2	E7	ZNF207	E6	CDK11B
E5	SLC4A2	E5B	PSMC2	E6	HDAC4	E7	AP2S1	E6	TAF1
E5	DNAJC11	E5B	SGTA	E6	PTMA	E7	SLAIN2	E6	BGN
E5	MMP14	E5B	RPS27	E6	INTS10	E7	ABHD10	E6	FGFR3
E5	TMEM184C	E5B	STT3B	E6	PSMD13	E7	TJP2	E6	PRKACB
E5	DNAJB12	E5B	SURF4	E6	YIPF3	E7	JUN	E6	CDK2
E5	ABCC1	E5B	PSMD4	E6	SURF4	E7	CDKN1A	E6	GRK2
E5	FZD6	E5B	TFRC	E6	RTL8C	E7	PAK2	E6	PTPN4
E5	BCL2L13	E5B	ACBD3	E6	KRT8	E7	MAP2K3	E6	PML
E5	RPL21	E5B	TMPO	E6	UNC119B	E7	CDKN1B	E6	AKT2
E5	EGFR	E5B	TIMM50	E6	SIK3	E7	RPL21	E6	GRK5
E5	ERBB2	E5B	COPB2	E6	PRKAR1A	E7	SRP14	E6	RDX
E5	MT-ATP6	E5B	CALR	E6	WDYHV1	E7	PRKAA2	E6	FBN1
E5	GPRC5B	E5B	SSR4	E6	VKORC1	E7	TALDO1	E6	CSK
E5	FADS2	E5B	COPE	E6	KRT18	E7	PCGF6	E6	PRKCI
E5	PSEN2	E5B	SGPL1	E6	SFI1	E7	CA8	E6	TEC
E5	MTCH1	E5B	PSMC4	E6	YDJC	E7	SERPINB1	E6	NOTCH1

E5	SIDT2	E5B	EDEM3	E6	MRPS11	E7	CLK2	E6	NEDD4
E5	PIGU	E5B	SCD	E6	RAB33B	E7	SCNM1	E6	YAP1
E5	RMDN3	E5B	HSD17B12	E6	HNRNPR	E7	EIF4A1	E6	MAPKAPK2
E5	EMC2	E5B	PLOD1	E6	VPS26B	E7	DNAJA1	E6	MCM2
E5	CANX	E5B	KCT2	E6	BAG3	E7	UQCRC1	E6	CDK7
E5	EMD	E5B	SPTLC2	E6	RPLP0	E7	ELF1	E6	BLK
E5	FZD2	E5B	SPTLC1	E6	GSTK1	E7	TOR4A	E6	PRELP
E5	TMEM165	E5B	ITGB1	E6	SUPT4H1	E7	CBS	E6	NEK2
E5	GPR176	E5B	TM9SF1	E6	C1D	E7	LNX2	E6	KPNA1
E5	MOGS	E5B	PLXNB2	E6	CASK	E7	ELANE	E6	CRIP2
E5	LAMTOR1	E5B	UBAC2	E6	GM2A	E7	KRT16	E6	LIMK1
E5	SLC1A5	E5B	PGRMC2	E6	RPL23	E7	DNAJA3	E6	UBE2I
E5	TAPBP	E5B	TTC13	E6	PRPF31	E7	CHUK	E6	JAG1
E5	RDH11	E5B	GLIPR1	E6	DYNC1H1	E7	ZNF408	E6	FBLN2
E5	SCN9A	E5B	NDUFA4	E6	OXSM	E7	MRFAP1L1	E6	IRF9
E5	HSD17B12	E5B	GDAP1	E6	GALNT5	E7	SARS2	E6	MAP2K1
E5	SERINC1	E5B	LAPTM4A	E6	CASP10	E7	RBMXL2	E6	TEK
E5	SEC62	E5B	MAVS	E6	RACK1	E7	RPL31	E6	MAP3K10
E5	REEP5	E5B	ATP2C1	E6	COX7C	E7	FUBP3	E6	NOTCH2
E5	HM13	E5B	GRAMD2B	E6	CNP	E7	MIF	E6	PRKCQ
E5	OSTC	E5B	SLC7A5	E6	TUBA1C	E7	REL	E6	PTPN12
E5	TMPO	E5B	SLC25A11	E6	RPS6	E7	KIF20A	E6	PRKCZ
E5	ABCD3	E5B	SEC11A	E6	THRAP3	E7	CUL1	E6	PRKCD
E5	SLC30A1	E5B	QSOX1	E6	SNX27	E7	RBX1	E6	PTPN11
E5	MAVS	E5B	USMG5	E6	FAM50B	E7	FARS2	E6	TJP1
E5	RFTN1	E5B	STX4	E6	RASD1	E7	ISCA2	E6	BAX
E5	IKBIP	E5B	TAP1	E6	DLG4	E7	E2F6	E6	BCL2L1
E5	ATL3	E5B	NOTCH2	E6	TSEN54	E7	HMGN2P46	E6	MCL1

E5	VEZT	E5B	SLIRP	E6	HNRNPDL	E7	AES	E6	DMPK
E5	SLC38A10	E5B	SEC61B	E6	PKN1	E7	IGFBP3	E6	EFEMP1
E5	LNP	E5B	ASPH	E6	NACA	E7	FOXM1	E6	BPTF
E5	CEPT1	E5B	ERGIC1	E6	CIZ1	E7	TMED10	E6	PTPN13
E5	IMMT	E5B	PSMC1	E6	NDUFB11	E7	RNF2	E6	PAK1
E5	ATP2A2	E5B	PON2	E6	CCDC120	E7	WDYHV1	E6	PAK2
E5	TMEM179B	E5B	ESYT2	E6	RPSA	E7	ZMYND10	E6	STK3
E5	BCAP31	E5B	CDIPT	E6	DMAC2	E7	DDX42	E6	SNTA1
E5	CREB3	E5B	NPLOC4	E6	TNFRSF10A	E7	DIS3	E6	ROCK1
E5	PLD3	E5B	ATAD3A	E6	KPNA2	E7	PHC2	E6	CAMK2B
E5	HACD3	E5B	RTN4	E6	ILVBL	E7	FLNB	E6	CAMK2G
E5	TMEM175	E5B	BAG2	E6	TMSB10	E7	AP3B1	E6	FHL1
E5	TRPV4	E5B	FAR1	E6	GJA1	E7	GALNT5	E6	PRKG1
E5	GPX8	E5B	ADAM10	E6	C7orf50	E7	JUND	E6	IL16
E5	PON2	E5B	FTH1	E6	LCN2	E7	CREBBP	E6	CASP8
E5	SLC30A7	E5B	PRAF2	E6	RTP5	E7	SVIL	E6	PRKD1
E5	RPS27L	E5B	PHB2	E6	GAS6	E7	AK3	E6	RPS6KA2
E5	ATP5F1	E5B	APOE	E6	TPT1	E7	RUFY3	E6	RPS6KA1
E5	COX15	E5B	DDOST	E6	ELOC	E7	PPP1R18	E6	SLC9A3R2
E5	APMAP	E5B	SLC4A2	E6	KRT13	E7	GABPB2	E6	MYLK
E5	TM9SF3	E5B	RPN2	E6	COG6	E7	WWC2	E6	STK11
E5	GCN1	E5B	MFGE8	E6	YWHAB	E7	CDK4	E6	MAPKAPK3
E5	LAPTM4A	E5B	FADS2	E6	GYS1	E7	BEND7	E6	LRRK2
E5	TBL2	E5B	CLPTM1	E6	ANXA3	E7	TGM2	E6	MAGI3
E5	VAPA	E5B	GPD2	E6	GAPDH	E7	ATP5O	E6	CDC42BPA
E5	TMEM158	E5B	CDS2	E6	FBLN1	E7	AIDA	E6	RAP1GAP2
E5	MLEC	E5B	IGF2R	E6	KRT15	E7	L3MBTL2	E6	MAST2
E5	PIEZO1	E5B	MPZL1	E6	KRT4	E7	HNRNPM	E6	TAOK1
E5	ARV1	E5B	CNP	E6	MAN1B1	E7	PPP1CA	E6	MAGI2

E5	GOLT1B	E5B	ERLIN1	E6	EEF1B2	E7	ZNF417	E6	WWC1
E5	FATE1	E5B	PDGFRB	E6	THOC5	E7	PPP2R1A	E6	PIAS4
E5	SPNS1	E5B	SEC22B	E6	MED1	E7	OAZ2	E6	MINK1
E5	SCAMP4	E5B	MGST1	E6	MKS1	E7	RING1	E6	LNX1
E5	MRGPRF	E5B	SLC25A12	E6	FARSA	E7	GSTO2	E6	NEK9
E5	CDKAL1	E5B	EGFR	E6	RPL39	E7	TOX4	E6	SETD7
E5	PBXIP1	E5B	MT-CO2	E6	MRPS33	E7	CARMIL3	E6	DLG3
E5	STT3B	E5B	DNAJA1	E6	ATF4	E7	MIS18BP1	E6	IRF7
E5	MPV17L2	E5B	ABCC1	E6	VEGFB	E7	LNX1	E6	AURKB
E5	YIF1B	E5B	FZD6	E6	COL6A1	E7	ENO1	E6	NEURL4
E5	PIGG	E5B	GREM1	E6	JAK1	E7	ZBTB16	E6	NEDD4L
E5	RABL3	E5B	GPC1	E6	TEAD4	E7	AP2A2	E6	PASK
E5	PIGO	E5B	ATP1A1	E6	TEAD2	E7	PKM	E6	PPP1R9B
E5	DHRS7	E5B	ICMT	E6	PKM	E7	HLA-A	E6	NELL2
E5	TMEM35A	E5B	ADCY6	E6	FGFBP1	E7	HK1	E6	NOTCH4
E5	COX20	E5B	STOM	E6	M6PR	E7	FOS	E6	PRMT1
E5	WLS	E5B	RPL37A	E6	LGALS1	E7	CCNE2	E6	TLR10
E5	CD44	E5B	HMOX2	E6	FLNA	E7	PAK4	E6	PRKD2
E5	SGPL1	E5B	AXL	E6	SELENOW	E7	AP2A1	E6	WWP1
E5	RTN4	E5B	LOX	E6	TBL1XR1	E7	SAP130	E6	TAOK3
E5	ERGIC1	E5B	MICAL2	E6	PFKM	E7	TMEM50A	E6	HIPK2
E5	PIGT	E5B	PTPN1	E6	PSMC3	E7	HNRNPF	E6	TP63
E5	SURF4	E5B	UFD1	E6	SRPK1	E7	HDAC1	E6	EPB41L1
E5	TMEM43	E5B	RAP1A	E6	TRAPPC6A	E7	MPHOSPH8	E6	RXFP1
E5	RFT1	E5B	ENG	E6	ANKRD13A	E7	EIF4B	E6	SGK2
E5	TMEM259	E5B	CAVIN3	E6	TSTA3	E7	FLNA	E6	NEK6
E5	ANO6	E5B	CHRM2	E6	CD63	E7	JAK1	E6	LATS2
E5	KIAA2013	E5B	JAK1	E6	LAMB3	E7	USF1	E6	NDUFA13
E5	THEM6	E5B	SLC3A2	E6	MRPL53	E7	GOT2	E6	STK26

E5	SSR4	E5B	SRPRA	E6	KRT7	E7	UQCR11	E6	TNFRSF10D
E5	SCFD1	E5B	ATP2B4	E6	GEMIN7	E7	STAMBPL1	E6	TJP2
E5	ERLEC1	E5B	GJA1	E6	SLC3A2	E7	DDX17	E6	BAZ2B
E5	PKD2	E5B	CSPG4	E6	KLK5	E7	HNRNPC	E6	MCM8
E5	ESYT2	E5B	IKBIP	E6	NT5E	E7	HNRNPH1	E6	TNIK
E5	PTRH2	E5B	RPS13	E6	PSMD6	E7	SKP2	E6	PPP1R9A
E5	LPAR1	E5B	NCEH1	E6	DCTN1	E7	HSPA8	E6	CAMK2A
E5	PIGK	E5B	CAVIN1	E6	TNFRSF1A	E7	PLCB3	E6	TLR6
E5	DNAJC16	E5B	SLC27A1	E6	INF2	E7	MT-CYB	E6	EPB41L3
E5	COMT	E5B	PGRMC1	E6	ETF1	E7	IGFBP4	E6	PTPN22
E5	CHRM2	E5B	HAX1	E6	CSPP1	E7	MT-CO1	E6	MAP3K2
E5	S100A16	E5B	TMX3	E6	HSP90B1	E7	MT-CO2	E6	MAP3K4
E5	DHCR24	E5B	MBOAT7	E6	COL6A2	E7	FUS	E7	SGK1
E5	SARAF	E5B	CHP1	E6	EBP	E7	MT-CO3	E7	CHD1
E5	ATP5J	E5B	SLC27A4	E6	ADH5	E7	POU5F1	E7	MAP2K7
E5	SLC22A18	E5B	IMMT	E6	SNRPB	E7	EAF1	E7	CHEK1
E5	TMCO3	E5B	LRP10	E6	KRT83	E7	MT-ND6	E7	PPEF1
E5	TMEM205	E5B	PREB	E6	RIPK1	E7	AP2B1	E7	IKBKB
E5	PHB2	E5B	MEGF8	E6	PTHLH	E7	GOT1	E7	CASK
E5	SLC3A2	E5B	GALNT5	E6	HLA-C	E7	MT-ND5	E7	AURKA
E5	TMPPE	E5B	MTCH2	E6	NUP153	E7	LMNA	E7	TRIM24
E5	CLCC1	E5B	ABLIM3	E6	GPR87	E7	SFRP1	E7	HDAC3
E5	CSPG4	E5B	SGCD	E6	RAB33A	E7	FOSL2	E7	MAP3K7
E5	TBXA2R	E5B	CLU	E6	TSC2	E7	HDAC2	E7	FOXO3
E5	SLC30A9	E5B	SLC2A1	E6	FASN	E7	EXOC3-AS1	E7	AIRE
E5	TMX2	E5B	SARAF	E6	PXN	E7	MT-ND4	E7	KALRN
E5	RAP1A	E5B	SPARC	E6	CD97	E7	SELENOW	E7	SMARCA5
E5	PDIA3	E5B	SLC22A18	E6	ENO1	E7	MT2A	E7	KDM1A
E5	F2R	E5B	PBXIP1	E6	PSMD8	E7	JUNB	E7	JAK2
E5	ATP2B4	E5B	CREB3L1	E6	DUS1L	E7	PTHLH	E7	ROCK2

E5	UBAC2	E5B	ZFPL1	E6	TMEM205	E7	TARDBP	E7	TRIO
E5	S100A13	E5B	ERLIN2	E6	RPL3	E7	CAPNS1	E7	ELP1
E5	IGF2R	E5B	F2R	E6	CTSA	E7	COX7C	E7	MAP4K4
E5	ATP5O	E5B	GRAMD1A	E6	RPS9	E7	CDK5	E7	CHEK2
E5	SERPINE2	E5B	TMEM158	E6	RPL13A	E7	MT1F	E7	NSD2
E5	FITM2	E5B	RMDN3	E6	RPL10	E7	L3MBTL3	E7	ABL1
E5	PIGS	E5B	TM9SF3	E6	TXN	E7	PRNP	E7	PLAU
E5	MXRA7	E5B	SRPRB	E6	BRCA1	E7	TBL1Y	E7	AGT
E5	RNF170	E5B	CCPG1	E6	MTA2	L1	HSPA8	E7	NRAS
E5	PGRMC1	E5B	ALG1	E6	IFI27	L1	TNPO1	E7	HLA-DRA
E5	OCIAD1	E5B	AMFR	E6	UTRN	L1	IPO5	E7	HLA-DRB1
E5	KCT2	E5B	TRHDE	E6	DLK2	L1	KPNA2	E7	FGB
E5	KCTD12	E5B	PCDHGB5	E6	ALDH1A3	L1	KPNB1	E7	NR3C1
E5	CSF1R	E5B	PSMD1	E6	TRAM1	L2	FTL	E7	TUBB4A
E5	ATAD1	E5B	SSR3	E6	RPS16	L2	KPNA2	E7	HLA-DPB1
E5	SRPRA	E5B	DNAJC16	E6	SCRIB	L2	KPNB1	E7	PRKCG
E5	RER1	E5B	WRNIP1	E6	PRIM2	L2	GADD45GIP1	E7	PRKCB
E5	HLA-A	E5B	ESYT1	E6	ZYX	L2	AUP1	E7	LCK
E5	PTPN1	E5B	SQOR	E6	TNS3	L2	PATZ1	E7	FYN
E5	ATAD3A	E5B	RAB34	E6	SLCO3A1	L2	THAP11	E7	PGR
E5	TMX3	E5B	SNX14	E6	TMEM214	L2	SLC6A8	E7	CAPN1
E5	SQOR	E5B	AFG3L2	E6	DAB2IP	L2	TINAGL1	E7	TUBB
E5	TNFSF4	E5B	TBL2	E6	HNRNPA3	L2	PFDN5	E7	YES1
E5	CERS2	E5B	CHCHD3	E6	SAT1	L2	IPO5	E7	LYN
E5	TMEM38B	E5B	MXRA8	E6	RPL21	L2	KAT5	E7	RHOC
E5	NCEH1	E5B	RDH14	E6	LAMTOR1	L2	STK11	E7	NR3C2
E5	PTGS1	E5B	TMEM30A	E6	NT5DC1	L2	GNB2	E7	HSP90AB1

E5	USMG5	E5B	SLC38A10	E6	KANK2	L2	MAN2B1	E7	COL4A2
E5	GJA1	E5B	RNF170	E6	NLRC3	L2	TNPO1	E7	GSTP1
E5	TMEM214	E5B	TMEM165	E6	STEAP3	E6	SSBP	E7	AR
E5	ATP5H	E5B	MYOF	E6	RPL6	E6	P53	E7	RRAS
E5	SEC61A1	E5B	GGT7	E6	VIM	L2	SNX17	E7	THRA
E5	DIRC2	E5B	RAB18	E6	TSTD2	E1	WDR48	E7	THRB
E5	CAVIN1	E5B	DPP7	E6	SPOUT1	E6	NEUL4	E7	VDR
E5A	VAPB	E5B	CDCP1	E6	FKBP15	E8^E2C	IDE	E7	SRC
E5A	VAPA	E5B	RETSAT	E6	COPG1	E2	UBR5	E7	HLA-E
E5A	TMEM181	E5B	PHTF1	E6	RPS13	E6	CWC15	E7	HLA-DRB4
E5A	CANX	E5B	PCDH18	E6	YLPM1	E1	PELP1	E7	HSP90B1
E5A	PTDSS2	E5B	VKORC1	E6	RPS11	E2	GKAP1	E7	POU2F1
E5A	ESYT1	E5B	ATP13A1	E6	LPCAT2	E1	WDR20	E7	RAC2
E5A	IGF2R	E5B	LNP	E6	SLC4A5	E7	AP1M1	E7	ATF2
E5A	VKORC1	E5B	ERBB2	E6	TLR7	E7	PTN14	E7	NME1
E5A	MLEC	E5B	TOR4A	E6	LAMA3	E1	CDK1	E7	FER
E5A	COPB2	E5B	SLC25A13	E6	EIF1	E5	BAG6	E7	PRKACA
E5A	TMEM175	E5B	DNAJC11	E6	HERC2	E5	MBRL	E7	EIF2AK2
E5A	MGST1	E5B	SEC63	E6	HGS	E7	MAP2	E7	CSNK2A2
E5A	STT3A	E5B	SLC25A22	E6	DCAF6	E1	UBP1	E7	RXRA
E5A	SEC61B	E5B	PCYOX1	E6	TPRA1	E5	DNJB1	E7	RAB3B
E5A	ATP2C1	E5B	SACM1L	E6	CMTM1	E1	AN32A	E7	CDK11B
E5A	COPA	E5B	TRPV2	E6	FHL2	E6	PSD11	E7	TAF1
E5A	RNF170	E5B	SFXN3	E6	PDZD11	E7	CREB1	E7	NME2
E5A	VMP1	E5B	ITM2C	E6	SLC44A2	E7	RM14	E7	PRKACB
E5A	SLC38A2	E5B	APMAP	E6	RPS27	E7	ABHDA	E7	NR4A1
E5A	SLC35A2	E5B	ARMCX3	E6	PROM2	E1	RAGP1	E7	TUBG1
E5A	GLIPR1	E5B	ABCB6	E6	KPNB1	E1	CCNB1	E7	IGFBP5
E5A	CLU	E5B	QPCTL	E6	LARP1B	E7	RT12	E7	TNC
E5A	RAB5C	E5B	GLG1	E6	RFC5	E7	PAF15	E7	GRK2
E5A	SLC2A1	E5B	TMEM109	E6	ZMIZ1	E1	ECHA	E7	MAPK3

E5A	SERPINE2	E5B	TOR1AIP2	E6	USP2	E1	TEX10	E7	MAPK1
E5A	RABL3	E5B	DNAJB14	E6	RPL7A	E5	CKAP4	E7	KDM5A
E5A	TMEM184C	E5B	TECR	E6	RPL5	E6	ECI2	E7	PML
E5A	YIF1B	E5B	SPNS1	E6	TMEM109	E5	DNJC7	E7	HLA-F
E5A	KDELR3	E5B	SH3BP4	E6	AP5S1	E1	UBP46	E7	AKT2
E5A	LNP	E5B	FAM8A1	E6	RPS12	L1	IMA4	E7	GRK5
E5A	SEC11A	E5B	RRBP1	E6	MBOAT7	E5	RPN1	E7	RORA
E5A	PIGS	E5B	OSTC	E6	PDZRN3	E5	UBL4A	E7	PPARG
E5A	FAS	E5B	CD44	E6	RPS5	E5	ATPB	E7	COL18A1
E5A	RAP1A	E5B	DNAJB9	E6	TMEM79	E7	SRC8	E7	PRKCI
E5A	GJA1	E5B	SUN1	E6	CHPF	L1	TCPH	E7	ACTR1B
E5A	MMP14	E5B	VKORC1L1	E6	VRK2	E1	AN32B	E7	ABL2
E5A	WLS	E5B	LRRC59	E6	PLK2	E5	DNJB4	E7	MAPK8
E5A	SGTA	E5B	ABCA1	E6	FLOT1	E8^E2C	BIRC6	E7	MAPK9
E5A	TMEM109	E5B	PDLIM7	E6	MAP1S	E1^E4	WDR6	E7	ATRX
E5A	YIPF3	E5B	PIEZO1	E6	BCAP31	E7	TPX2	E7	NSF
E5A	ACBD3	E5B	ERP44	E6	TMEM17	E5	STIP1	E7	CSNK1A1
E5A	TMEM35A	E5B	ENDOD1	E6	RIPOR1	E7	PTN21	E7	NR2C2
E5A	TMEM161A	E5B	STAU1	E6	RPL24	L1	IMA1	E7	MAPKAPK2
E5A	CHCHD3	E5B	RDH11	E6	ATP6V1H	E6	ECH1	E7	CLK1
E5A	AGK	E5B	COMT	E6	CDR2L	L2	PLEC	E7	GSK3A
E5A	CHRM2	E5B	SELENOK	E6	RXRB	E7	DHSA	E7	GSK3B
E5A	DDOST	E6	RPL4	E6	IDH3G	E5	F10A1	E7	CDK7
E5A	ESYT2	E6	BAK1	E6	CDKN1A	E7	AAMP	E7	CDK9
E5A	FAF2	E6	CLUH	E6	PEF1	L2	MAGD2	E7	RPS6KA3
E5A	TRAM1	E6	TP73	E6	RPS3	E7	TCPE	E7	NEK2
E5A	ATP1A1	E6	TYK2	E6	BAIAP2	E1	ECHB	E7	MAP2K6
E5A	MXRA8	E6	SIK1	E6	DAG1	E5	UBQL4	E7	LIMK1
E5A	HM13	E6	SERPINB5	E7	THAP11	E6	WRIP1	E7	NR1H2
E5A	FAM8A1	E6	MAP2K2	E7	PFKP	L1	PITM2	E7	VCP
E5A	PTTG1IP	E6	SERTAD1	E7	CBX4	L2	BRE1A	E7	HDAC4

E5A	TMPO	E6	LDHA	E7	E2F2	E1	H1T	E7	ACTB
E5A	DNAJC16	E6	SCAMP2	E7	SAMHD1	E7	DEST	E7	RAC3
E5A	CPT1A	E6	HSPA5	E7	USH1C	E7	CREM	E7	CDC42
E5A	F2R	E6	EP300	E7	HMGN2	L1	RRP12	E7	RAB8A
E5A	DHCR24	E6	KDM1A	E7	E2F1	L1	RBM28	E7	RAB10
E5A	SEC62	E6	PCDH11X	E7	EXOSC1	E2	CCHCR	E7	RAB14
E5A	DAD1	E6	GEM	E7	RPS27L	L1	TITIN	E7	ACTR3
E5A	GGCX	E6	APP	E7	EIF3F	E7	MCM5	E7	ACTR2
E5A	SCN9A	E6	9-Sep	E7	INO80B	E1	WDR18	E7	ACTR1A
E5A	GPR176	E6	EIF5	E7	GALE	E1^E4	HMGN1	E7	RHOA
E5A	ARL1	E6	SNTB2	E7	AP2M1	E8^E2C	ZZEF1	E7	PSMC6
E5A	LOX	E6	PRKAA2	E7	MAP7	E1	AN32C	E7	ACTA2
E5A	HMOX2	E6	DLG1	E7	PCBP2	E2	1433G	E7	RAP1A
E5A	SLC1A5	E6	TM4SF1	E7	EIF3I	E2	PNMA2	E7	UBE2D2
E5A	TAPBP	E6	RAB15	E7	CEP44	E2	CSK21	E7	RAC1
E5A	SFXN3	E6	MT-CO1	E7	CTTN	E2	RUVB2	E7	ACTG1
E5A	BDKRB2	E6	SIPA1L2	E7	GAS6	E2	LIPA1	E7	UBE2L3
E5A	SLC7A11	E6	MT-CYB	E7	CSNK2A1	E6	CC85C	E7	TUBB4B
E5A	SLC22A18	E6	NAGK	E7	BLOC1S6	E1	DMWD	E7	HLA-DRB3
E5A	PDGFRB	E6	C3	E7	MGA	E1	KEAP1	E7	RHOG
E5A	STOM	E6	MT-ATP6	E7	CHERP	L2	QCR2	E7	CDK6
E5A	SLC35E1	E6	PTPN3	E7	CAVIN3	E7	PDD2L	E7	IRF9
E5A	ABCC1	E6	TMEM106C	E7	CAVIN1	E7	MELK	E7	MAP2K1
E5A	BSCL2	E6	MESDC2	E7	AP1M2	L1	ZCHC3	E7	ERCC6
E5A	PKD2	E6	MYOF	E7	PPP2CA	E5	XPO2	E7	TAP1
E5A	BAG2	E6	LOX	E7	SQOR	E5	ELOV5	E7	PRKCQ
E5A	HAX1	E6	MT-CO3	E7	CUL2	E5	DHCR7	E7	PRKCZ
E5A	ATP5H	E6	EML2	E7	ZGPAT	E5	UN45A	E7	PRKCD
E5A	SURF4	E6	MT-CO2	E7	TUBA1C	E8^E2C	HECD3	E7	PPARA
E5A	CNP	E6	MAP4	E7	RPL18	E6	PRS6A	E7	TLE5
E5A	S100A16	E6	TCHP	E7	STX8	L2	CMC2	E7	PPP3CA
E5A	SSR4	E6	RRBP1	E7	INTS14	L1	YTHD3	E7	ITK
E7	FOXO1	E5	TNPO2	E5	STK26	E2	HIST1H2AG	E2	KPNA2
E7	BPTF	E5	IKBKB	E5	TNIK	E2	RPS19	E2	HNRNPK
E7	CHD3	E5	AURKA	E5	ALK	E2	RPL34	E2	STK38

E7	ZBTB17	E5	CHUK	E5	MAP3K2	E2	RPS16	E2	MRPL47
E7	PAK1	E5	MAP3K7	E5	RIPK3	E2	RPS23	E2	PRMT5
E7	MAPK7	E5	RIPK2	E5	MAP3K4	E2	HIST1H2BC	E2	RPS9
E7	STK3	E5	JAK2	E2	DAXX	E2	RPL31	E2	RPL23A
E7	DNAJC3	E5	MAP4K4	E2	NPM1	E2	MRPS25	E2	ICT1
E7	NR5A1	E5	ABL1	E2	HNRNPC	E2	RPS17	E2	LBR
E7	ROCK1	E5	FOS	E2	RFC4	E2	RPL28	E2	MRPL40
E7	BIRC3	E5	HLA-DRA	E2	WDR5	E2	RPL12	E2	MRPL11
E7	PRPF4B	E5	HLA-DRB1	E2	SET	E2	RPS11	E2	MRPL24
E7	CAMK2B	E5	HLA-DPB1	E2	PCBP2	E2	RPS15	E2	ELAVL1
E7	CAMK2G	E5	PRKCG	E2	TUBB	E2	RPS24	E2	MRPL4
E7	CUL3	E5	JUN	E2	EEF1G	E2	RPL32	E2	MRPL1
E7	DYRK1A	E5	PRKCB	E2	HNRNPF	E2	RPS10	E2	MRPL44
E7	SPTAN1	E5	INSR	E2	EEF1A1	E2	RPL26	E2	MRPL15
E7	PRKG1	E5	LCK	E2	TUBA1B	E2	RPL27A	E2	HNRNPDL
E7	FHL2	E5	FYN	E2	DDX39B	E2	RPL13	E2	RPSA
E7	IRF3	E5	CDK1	E2	BYSL	E2	RPL14	E2	HNRNPA2B1
E7	PRKD1	E5	YES1	E2	HSPA8	E2	H1FX	E2	HNRNPH3
E7	RPS6KA1	E5	LYN	E2	PABPC1	E2	SNRPA	E2	MRPL39
E7	TAF1C	E5	RET	E2	XRCC6	E2	HIST1H1E	E2	RALY
E7	RHOH	E5	FGR	E2	PRPF6	E2	RPL7	E2	HNRNPA3
E7	MYLK	E5	CDK4	E2	MATR3	E2	RPL5	E2	HNRNPAB
E7	SMAD2	E5	SRC	E2	HNRNPU	E2	EXOSC7	E2	WDR77
E7	SMAD1	E5	HLA-DRB4	E2	SF3A1	E2	E2F6	E2	GTPBP10
E7	ITSN1	E5	PDGFRA	E2	USP11	E2	DNAJC9	E2	CSNK2A2
E7	STK11	E5	FER	E2	XRN2	E2	TFPT	E2	ILF2
E7	BATF	E5	PRKACA	E2	SF3B1	E2	RFC5	E2	MRPS27
E7	MAPK14	E5	PLCG1	E2	DHX9	E2	RPL6	E2	MRPL37
E7	MAPKAPK3	E5	EIF2AK2	E2	BNC2	E2	RPLP0	E2	RBMX

E7	MAPK6	E5	HLA-DPA1	E2	SUGP2	E2	RFC2	E2	HNRNPD
E7	HIF1A	E5	ERBB3	E2	ADNP	E2	RFC3	E2	MRPL38
E7	LRRK2	E5	FGFR3	E2	SNRPD1	E2	HSU53209	E2	HNRNPH1
E7	OBSCN	E5	CBL	E2	SNRPD2	E2	UCHL5	E2	LUC7L2
E7	MAST2	E5	PRKACB	E2	SNRPD3	E2	ACTL6A	E2	ATP5A1
E7	TUBA1A	E5	JAK1	E2	SF3B6	E2	NR2F1	E2	C11orf84
E7	RHOU	E5	CDK2	E2	SRP14	E2	RPL4	E2	U2AF2
E7	TAOK1	E5	MAPK3	E2	BUD31	E2	YBX1	E2	HNRNPL
E7	MINK1	E5	ATP6V0C	E2	NCBP2	E2	SMARCB1	E2	HDAC1
E7	NEK9	E5	MAPK1	E2	MAGOH	E2	KRR1	E2	DMAP1
E7	JDP2	E5	EPHA2	E2	SAP18	E2	WDR18	E2	NOP58
E7	HTRA1	E5	EPHB2	E2	PPIH	E2	INO80B	E2	SYNCRIP
E7	RORB	E5	HLA-F	E2	PPIL3	E2	RUVBL2	E2	HSPA9
E7	KAT2A	E5	AXL	E2	PPIL1	E2	RUVBL1	E2	ZNF326
E7	IRF7	E5	PTGS2	E2	SRSF3	E2	GTF3C5	E2	ILF3
E7	USP9X	E5	KDR	E2	RBM8A	E2	CDYL	E2	HNRNPUL2
E7	CUL5	E5	MAP2K2	E2	CFAP20	E2	RBBP5	E2	DDX21
E7	SH3KBP1	E5	CDKN1A	E2	SNRPB2	E2	RBM39	E2	GTF3C3
E7	AURKB	E5	CSK	E2	SNRPB	E2	DDX3X	E2	BRCA1
E7	RHOV	E5	PRKCI	E2	SNRPA1	E2	ACTR8	E2	CDC20
E7	PASK	E5	MATK	E2	CXorf56	E2	MTA2	E2	CFLAR
E7	RSF1	E5	TEC	E2	THOC6	E2	HP1BP3	E2	CPSF4
E7	SORBS1	E5	ZAP70	E2	TRA2B	E2	PATZ1	E2	CUL3
E7	TLR10	E5	SYK	E2	ISY1	E2	ASH2L	E2	EIF6
E7	HDAC8	E5	CDKN1B	E2	ACTB	E2	MTA1	E2	GNB2L1
E7	NSD3	E5	ATP5PO	E2	EIF4A3	E2	NOL9	E2	KDM5C
E7	PRKD2	E5	GSK3B	E2	SRSF6	E2	NKRF	E2	PLK1
E7	TAOK3	E5	CDK7	E2	RNPS1	E2	KDM1B	E2	SFRS1
E7	HIPK2	E5	BLK	E2	TUBA1C	E2	BAP1	E2	SFRS2
E7	CLK4	E5	NEK2	E2	VIM	E2	WHSC1L1	E2	SFRS7
E7	RHOF	E5	LIMK1	E2	PRPF19	E2	GTF3C4	E2	SKP2
E7	MKNK2	E5	HLA-DRB3	E2	PLRG1	E2	LAS1L	E2	SNRNP70
E7	NEK6	E5	CDK5	E2	SF3A3	E2	NOP2	E2	TAF7
E7	TUBG2	E5	MAP2K1	E2	SF3A2	E2	CTCF	E2	TRA2B

E7	STK26	E5	MAP3K10	E2	PUF60	E2	GTF3C2	E2	TRAF5
E7	HDAC6	E5	PRKCQ	E2	SNW1	E2	DDX24	E2	TRAF6
E7	BAZ2B	E5	MST1R	E2	DDX17	E2	KIF11	E2	TNPO3
E7	ANAPC2	E5	PTK2	E2	DDX41	E2	GTF2I	L2	ANXA2
E7	RPS6KA6	E5	PRKCZ	E2	DHX15	E2	INO80D	E2	CDH1
E7	TNIK	E5	PRKCD	E2	HNRNPM	E2	BCLAF1	E2	CFLAR
E7	HDAC9	E5	BTK	E2	NCBP1	E2	ZFR	E2	EIF6
E7	ZMYND8	E5	BAX	E2	IK	E2	SMC6	E2	GNB2L1
E7	COL17A1	E5	DDR1	E2	SRPK1	E2	UBE2O	E2	GPS2
E7	VPS4A	E5	PAK1	E2	THOC1	E2	BAZ1B	E2	hSNF5
E7	CAMK2A	E5	MAPK7	E2	CRNKL1	E2	INO80	E2	PARP
E7	USP15	E5	PAK2	E2	ACIN1	E2	SMARCA2	E2	PCAF
E7	CD2AP	E5	ILK	E2	SRPK2	E2	SMARCA4	E2	SKP2
E4	KRT18	E5	PRKG1	E2	ZCCHC8	E2	ASXL2	E2	SFRS10
E4	CDK1	E5	PTK2B	E2	CDC5L	E2	PBRM1	E2	SMCX
E4	CDK4	E5	PRKD1	E2	PNN	E2	PSMD4	E7	CENPC
E4	CDK2	E5	ERBB4	E2	TRIM28	E2	GTF3C1	E8^E2C	CHD6
E4	MAPK3	E5	RPS6KA1	E2	XAB2	E2	CHD4	E2	GRIP1
E4	MAPK1	E5	MYLK	E2	SKIV2L2	E2	NUMA1	E2	PLAGL1
E4	MAPK8	E5	NTRK3	E2	EFTUD2	E2	BAZ2A	L1	L2
E4	CLK1	E5	MAPK14	E2	SF3B3	E2	ANKRD17	L2	ACTB
E4	CLK2	E5	NTRK2	E2	PAXBP1	E2	RIF1	E1	H1-1
E4	CDK7	E5	MAPK6	E2	SMARCA1	E2	PRKDC	E7	RB1
E4	SRPK2	E5	MICB	E2	SF3B2	E2	TRRAP		
E4	CDK6	E5	LRRK2	E2	THRAP3	E2	APCS		
E4	CDK5	E5	MAST2	E2	ZC3H18	E2	PARP1		
E4	PRPF4B	E5	TAOK1	E2	DHX8	E2	RBM25		
E4	MYLK	E5	MINK1	E2	DHX38	E2	SRRT		
E4	MAPK14	E5	NEK9	E2	AQR	E2	RPS3		

E4	HIPK2	E5	WNK4	E2	SNRNP200	E2	TMPO		
E4	CLK4	E5	WNK3	E2	PRPF8	E2	SPIN1		
E5	SGK1	E5	PRKD2	E2	RPLP2	E2	HNRNPA1		
E5	MAP2K7	E5	TAOK3	E2	RPL35A	E2	PPP2CA		
E5	CHEK1	E5	NEK6	E2	RPL30	E2	YY1		

Table S2: Pathway enrichment analysis of clusters 1 and 2 in HPV-Human protein interaction network

Cluster	Gene names	Pathway
**1**	ZZEF1, IDE, BIRC6, HECD3, CHD6, CNTN6	Apoptosis, Ubiquitin mediated proteolysis, Alzheimer disease
**2**	HSPA8, PPIB, KPNA2, TNPO1, KPNA1, KPNB1, IPO5	Influenza A, Legionellosis, Antigen processing and presentation, Longevity regulating pathway, Toxoplasmosis, Measles
